# Absence of genetic selection in a pathogenic *Escherichia coli* strain exposed to the manure-amended soil environment

**DOI:** 10.1371/journal.pone.0208346

**Published:** 2018-12-07

**Authors:** Allison M. Truhlar, Thomas G. Denes, Keiran K. Cantilina, Selene K. Leung, M. Todd Walter, Anthony G. Hay

**Affiliations:** 1 Department of Biological and Environmental Engineering, Cornell University, Ithaca, New York, United States of America; 2 Department of Food Science, University of Tennessee, Knoxville, Tennessee, United States of America; 3 Department of Microbiology, Cornell University, Ithaca, New York, United States of America; Purdue University, UNITED STATES

## Abstract

*Escherichia coli* that express curli are more common in subsurface soil drainage when manure is surface applied. However, it is unknown whether this arises from mutations in individual strains leading to curli expression or by selection for individuals already expressing higher levels of curli. To test this, we examined curli production in pathogenic *E*. *coli* O157:H7 EDL933 as a function of manure management. Five treatments were investigated: (1) soil only, (2) soil with surface-applied *E*. *coli* O157:H7 EDL933 Δ*stx*_1-2_ (EcO157), (3) soil with incorporated EcO157, (4) soil with surface-applied EcO157-inoculated manure, and (5) soil with incorporated EcO157-inoculated manure. EcO157 was reisolated from soils immediately after application and weekly thereafter for 8 weeks. EcO157 in the surface-applied treatments died faster than their incorporated treatment counterparts. Phenotypic assays revealed differences between treatments as well. Half of surface-applied manure reisolates from week 6 developed a mixed red and white colony morphology on Congo Red plates, indicating changes in curli production that were not seen in other treatments or times. In 37°C growth tests, week 6 reisolates from all treatments except soil surface-applied EcO157 left the lag phase at a significantly greater rate than week 0 isolates. We applied whole genome sequencing technology to interrogate the genetic underpinnings of these phenotypes. Surprisingly, we only found single-nucleotide polymorphisms in two of the 94 resequenced isolates from the different treatments, neither of which correlated with curli phenotype. Likewise, we found no differences in other genomic characteristics that might account for phenotypic differences including the count of gaps and the origin of discarded reads that failed to map to the parental strain. These results suggest there were no systematic genomic differences (i.e. individual-level selection) that correlated with time or treatment. We recommend future research focus on population-level selection of *E*. *coli* strains in the manure-amended soil environment.

## Introduction

Water contamination with pathogenic *Escherichia coli* (*E*. *coli*) has “high” health significance based on both the incidence and severity of outbreaks [1]. A minor component of the gut microbiota of many mammals, both commensal and pathogenic strains may coexist in the same environment [2]. Verotoxigenic *E*. *coli* (VTEC) strains produce Shiga toxin, which is responsible for hemolytic uremic syndrome, characterized by the destruction of red blood cells and acute renal failure [3]. *Escherichia coli* O157:H7 is a pathogenic VTEC serotype that significantly contributes to disease outbreaks in North America and worldwide [3,4].

In agricultural landscapes, *E*. *coli* sourced from manure application can contaminate surface waters through two major hydrologic routes: surface runoff and subsurface drainage [5]. Controlling for initial concentration, the primary factor influencing the availability of *E*. *coli* for transport is the die-off rate of the bacteria [6]. Some strains of *E*. *coli* O157:H7 have been found to persist in manure-amended soil for more than 200 days, although persistence is strain, soil, and moisture dependent [7]. Soil is an important reservoir of *E*. *coli* that can cause contamination of drinking water, vegetables, and recreational waters. In agricultural areas the installation of drainage ditches may increase the *E*. *coli* burden in surface waters by short-circuiting natural surface and groundwater flow pathways [8,9]. Therefore, manure management techniques to minimize the persistence of *E*. *coli*, and thereby decrease the risk of downstream contamination, are of great interest.

Attenuation of *E*. *coli* in the environment arises from a variety of different stressors that impact their survival, including nutrient limitation, exposure to UV radiation, and fluctuations in temperature, pH, and humidity [3]. Multiple studies have suggested that water availability is the principal environmental factor affecting *E*. *coli* survival in soil. In general, *E*. *coli* has reduced mortality in soils with greater water content [10–12]. Furthermore, wetting and drying cycles increase the mortality rate of *E*. *coli* [10]. Prolonged *E*. *coli* survival has also been positively correlated with the amount of bioavailable carbon in the soil [13,14], finer textured soils [14,15], lower soil temperatures [11,16,17], and neutral to alkaline soil pH [17]. One added variable in the agricultural soil environment is manure application method. Soils with incorporated or injected manure support prolonged *E*. *coli* survival compared to soils with surface applied manure [18].

Curli is the main proteinaceous component of many *E*. *coli* biofilms, the extra-cellular matrix that encases aggregated cells and thus promotes survival [19]. Furthermore, curli expression has been associated with numerous stimuli including temperature, osmolarity, and electron acceptor availability [20]. In a previous study, we demonstrated that *E*. *coli* were more likely to express curli if they were isolated from the subsurface drainage of soil receiving surface-applied manure than if they were isolated from the drainage of manure-incorporated soil [21].

This phenotypic observation left open the question of the mechanism behind the differing curli phenotypes. Specifically, was there an individual- or population-level shift in the gene pool? In other words, did individual strains mutate to become curli over-expressers, or were the curli expressers we isolated already over-expressing curli? To date, genetic analyses aimed at understanding how genomic differences might influence *E*. *coli* survival in soil targeted a handful of genes associated with expression of curli and stress adaptations. For instance, both inter- and intra-strain comparisons of *E*. *coli* O157:H7 indicate that an intact *rpoS* gene, which is a global regulator of the general stress response, is correlated with increased survival in soil without manure [22,23]. However, it is unknown whether natural variation in *rpoS* or any other gene is actively selected for in soil and at what level this potential selection occurs. Thus, the more general question we hoped to answer in this work was if mutations were occurring in individual strains.

This present study aimed to determine how different manure management techniques impact the phenotypic and genomic population structure of *E*. *coli* O157:H7 EDL933 Δ*stx*_1-2_. We chose to isolate one environmental stressor, desiccation. This sets the stage for future work to address the individual effects of other environmental stressors on both *E*. *coli* survival and curli phenotype, unlike our previous work where *E*. *coli* were exposed to sunlight and changes in temperature in addition to desiccation. We assessed single nucleotide polymorphisms (SNPs), insertions, and deletions in the genomes of 94 strains that were reisolated after 5–8 weeks in soil microcosms. Surprisingly, we found no evidence for individual level selection in the genomes of these isolates which suggests that population level selection may be more important than individual selection in determining curli phenotype and soil survival.

## Methods

### Microcosm setup

The study organism, *E*. *coli* O157:H7 EDL933 Δ*stx*_1-2_ (EcO157), is an isogenic mutant derivative of *E*. *coli* O157:H7 EDL933, which has a fully sequenced and annotated genome [24]. It lacks the Shiga toxins Stx_1_ and Stx_2_ and has added cassettes for resistance to kanamycin (Km) and chloramphenicol (Cm) [14]. Importantly, this EcO157 derivative has an indistinguishable survival curve from the wild type strain in soils [14].

Overnight cultures of EcO157 in lysogeny broth (LB) with Km (50 μg/ml) and Cm (25 μg/ml) were harvested by centrifugation at 4°C, washed three times with phosphate buffer, and re-suspended in sterile deionized water [14]. The number of cells per ml of suspension was estimated by determining the optical density at 600 nm (OD_600_) with a spectrophotometer. This value was used to calculate the volume of inoculant required to reach a final concentration of 1 x 10^7^ colony forming units (cfu) per gram dry weight of soil.

Soil (Langford channery silt loam) was collected from the upper 30 cm of a marginal agricultural field owned by Cornell University in upstate New York. The site is designated for experimental use, and the Principal Investigator granted permission to collect the soil (B.K. Richards, personal communication). All soil was air dried, sieved at 2 mm and homogenized. Bovine manure was collected from the Cornell University teaching dairy facility. Both soil and manure were stored at 4°C.

Soil properties characterized included clay, silt and sand content, water content, water holding capacity (WHC), percent carbon (C) and percent nitrogen (N). Manure properties characterized included water content, percent C and percent N. Clay, silt and sand content were determined according to the hydrometer method [25]. Water content and WHC were determined according to previously described methods [7]. Percent C and N analyses were conducted at the Cornell University Stable Isotope Laboratory (COIL; Ithaca, NY). The soil and manure characteristics are described in Table 1.

**Table 1:** Soil and manure characteristics.

	Sand (%)	Silt (%)	Clay (%)	Bulk density (g/cm^3^)	WHC (g water/g dry material)	Initial water content (g water/g dry material)	Nitrogen (%)	Carbon (%)
**Soil**	36	62	2	0.79	0.7	0.17	0.3	3.2
**Manure**						4.4	2.3	40

Five treatments were investigated: (1) Soil only, (2) soil with surface-applied EcO157, (3) soil with incorporated EcO157, (4) soil with surface-applied EcO157-inoculated manure, and (5) soil with incorporated EcO157-inoculated manure. Four replicates were created for each treatment.

Microcosms were established in 120 mL urine collection containers covered with perforated aluminum foil lids to permit modest drying. Twenty-four hours before the start of the experiment, the air-dried soil was adjusted to 60% WHC using sterile deionized water [13,14] and returned to storage at 4°C. Each soil microcosm received 100 g of the wetted soil, equal to about 3 cm depth in the container.

The inoculated manure consisted of a 25 g manure subsample combined with an appropriate volume of EcO157 culture. From this mixture, two grams plus the fraction of additional weight from the EcO157 culture were added to the inoculated manure-treated microcosms. This is equivalent to a manure application rate of 2% w/w, which was used in related experiments [21], and follows suggested manure application rates [26]. The manure was then either incorporated by mixing with a metal spatula into the microcosm soil or left on the surface. When left on the surface, the manure added a surface layer with depth less than 1 cm.

For the soil with surface-applied or incorporated EcO157 treatments, EcO157 culture plus a volume of sterile deionized water equal to the water content of the manure was added, and then either left on the surface of the soil or incorporated into the soil. For the soil-only (control) treatment, a volume of sterile deionized water equal to the total EcO157 culture and manure moisture volume was added.

The microcosms were maintained at 15°C for the course of the experiment using a temperature-controlled growth chamber. This temperature is representative of the growing season (April to October) average soil temperature at a depth of 8 inches in central New York [S1 Dataset]. The microcosms were weighed weekly, and sterile deionized water was added to return the microcosms to their initial water content [S2 Dataset]. A two-way analysis of variance (ANOVA) was used to determine whether the weekly percent water loss differed significantly between treatment or week, and if there was any significant interaction between these two variables. Weekly percent water loss was calculated as the weight of water added to return to pre-desiccation weight, divided by the pre-desiccation water weight, assuming 60% WHC.

### Microcosm sampling and *E*. *coli* enumeration

Sampling occurred immediately following microcosm set-up (week 0) and weekly thereafter. At each sampling event, two 0.5 g soil samples were taken from each microcosm. Half of the samples were immediately frozen at -80°C for later DNA extraction. The other half of the samples was used immediately for enumeration of colony forming units of EcO157.

For enumeration, 1 ml of 0.1% peptone buffer was added to each soil sample [14]. The samples were then vortexed 2 x 20 s [14]. The resulting mixture was subjected to 10-fold serial dilutions to concentrations determined in preliminary experiments. Ten μl of the 2 to 3 highest dilutions were plated in triplicate on LB agar plates supplemented with Km (50 μg/ml) and Cm (25 μg/ml) [27]. The plates were incubated overnight at 30°C, at which time the colonies were distinctly visible for enumeration. The incubation temperature was selected to preserve curli expression in the reisolates for future testing [4]. The enumeration results were expressed as log colony forming units of EcO157 per gram dry weight of soil.

From each microcosm, 6 EcO157 isolates (where possible) were selected at random from the enumeration plates. These were inoculated into 100 μl LB broth and grown at 37°C for 16 hrs. One hundred μl of 50% glycerol, 50% sterile deionized water solution was added to the overnight cultures to achieve a final glycerol concentration of 25%. The cell suspensions were frozen at -80°C for later use.

### Phenotypic assays

To investigate phenotypic differences between *E*. *coli* isolated from different treatments, 15 EcO157 isolates were randomly selected from each of the four treatments collected during week 0 and week 6, for a total of 120 isolates. The first assay consisted of plating the isolates on YESCA Congo Red (CR) agar and growing them at 30°C for seven days [21, 28]. The plates were inspected every 24 hrs for morphology differences, indicative of differences in curli. Specifically, red colonies indicate the production of curli, while white colonies indicate the absence of curli [28]. *Escherichia coli* PHL628 WT and *E*. *coli* PHL628 Δ*csgA*, which has a known curli deficiency, were used as visual controls [29].

The second assay investigated differences in growth rate to determine if the reisolates’ persistence might be due to an ability to respond faster to favorable growth conditions. It consisted of growing a randomly selected subset of the CR assay isolates (6 per treatment for both weeks 0 and 6) in LB broth at 37°C and 15°C. The OD_600_ was measured over a 15 hr period in 96-well plates using a spectrophotometer. A specific growth constant was calculated for each treatment under these conditions using the specific growth rate equation for bacteria (Eq 1) [30], where N_1_ and N_2_ are the optical density measured at times t_1_ and t_2_, respectively, and k is the specific growth rate (hr^-1^). A one-way ANOVA followed by Tukey’s honestly significant difference (HSD) post hoc test was used to determine whether the specific growth constant differed significantly between treatments.

$$ln\frac{N_{2}}{N_{1}}=k(t_{2}-t_{1})$$ Eq 1

### DNA sequencing and bioinformatics

The following distribution of EcO157 isolates from inoculated samples were selected for whole genome sequencing: 5 isolates collected on week 0, 24 isolates (six per treatment) collected on week 3, 48 isolates (12 per treatment) collected on week 6, and 18 isolates (6 per treatment, except soil surface-applied) collected on week 8. All isolates were randomly selected except for those collected during week 6. These were selected to include isolates with observed morphology differences in the CR agar test described above.

DNA was extracted using a QIAamp DNA minikit (Qiagen, Hilden, Germany) with the addition of an RNase A (100 mg/ml; Qiagen) treatment [31]. A Nextera XT DNA sample preparation kit (Illumina, Inc., San Diego, CA) was used to prepare the library, and 2x75 bp paired-end reads were obtained by sequencing the library on the Illumina NextSeq platform.

Single nucleotide polymorphisms (SNPs), insertions and deletions (INDELs) were called using both a reference-based and *de novo* detection method. For the reference-based method, EcO157 isolate reads were mapped against the chromosome and plasmid of *E*. *coli* O157:H7 str. EDL933 (GenBank accession nos. CP008957.1 and CP008958.1) using CLC Genomics Workbench (CLC Bio, Qiagen), following a previously described pipeline [32]. The Cortex variation assembler, cortex_var, version 1.0.5.14 [33,34], was used for the *de novo* variant detection (both SNP and insertion/deletion events) [35]. For both methods, SNPs with a minimal 50% coverage of the genome-wide average coverage (GAC), a minimal 50% variant coverage of the GAC, and minimum 95% alternative variant frequency were considered for analysis [31].

We performed several more tests to further explore possible genomic differences overlooked by the mapping-to-reference method used in CLC Workbench (S1 Fig). First, we extracted the reference genome coverage for each sequenced strain to identify all regions with no coverage. Within each strain, these regions were grouped based on gene annotation. The rates of occurrence of no-coverage regions in a given gene were compared using a Poisson model for count data, with the combination of week-treatment as the sole explanatory variable. This was done to accommodate the fact that the control occurred only in week 0 and therefore could not have week as a separate explanatory variable. The same was true for the soil, surface-applied treatment, which did not yield isolates in week 8.

Finally, since mapping to the reference resulted in a consistent number of reads that did not match to the reference, we investigated the content of these discarded reads, which might represent additional genetic material that the strains received through horizontal gene transfer from other bacteria present in the soil [36]. The metagenomics RAST server (MG-RAST) pipeline was used to annotate the discarded reads (S1 Fig) [37]. We then compared the total number of discarded reads that could be annotated with a predicted feature using a linear model with week-treatment as the sole explanatory variable, as explained above. The predicted features that were used in this comparison were the top 25 genera for the origin of the unmapped DNA (by frequency). Functional subsystems were also included, except for those that correlated with another subsystem (∝ = 0.01). The number of predicted features that met these criteria were rank-transformed to meet normality requirements. Additionally, specific annotations, normalized to the number of predicted features for a given strain, were individually fit to a linear regression with week-treatment as the explanatory variable. The *p*-values from these regressions were adjusted to account for multiple comparisons, using the Benjamini-Hochberg method [38].

## Results and discussion

### *Escherichia coli* survival in soil

**Fig 1:**
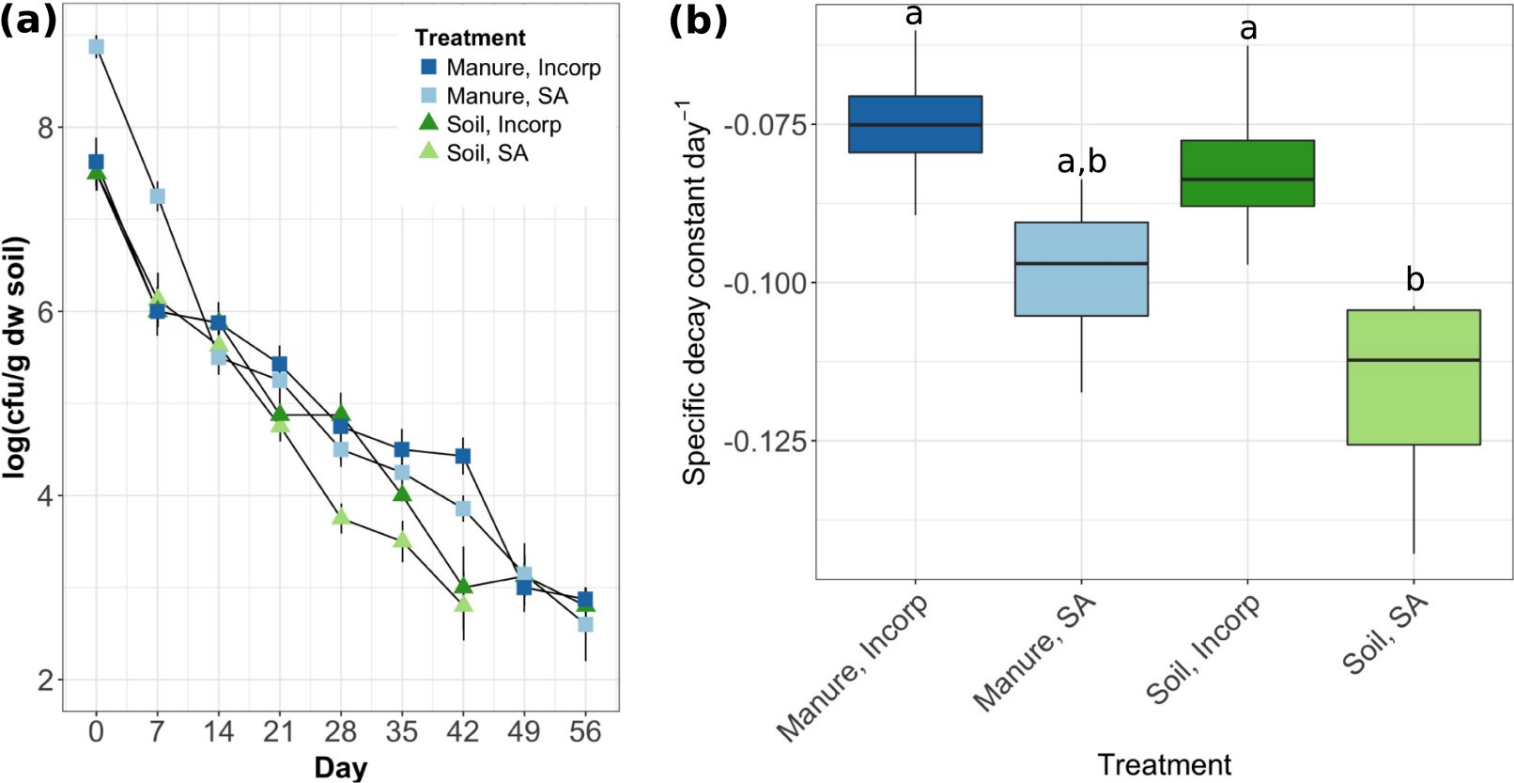
(a) Survival and (b) specific decay constants of *E*. *coli* O157:H7 EDL933 in soil microcosms with and without added manure (“Manure” and “Soil,” respectively). “Incorp” indicates mixing of the soil after *E*. *coli* application; “SA” indicates surface application (i.e., no mixing). Data represents an average +/- one standard deviation (1SD) of four replicates. Different letters indicate significant differences in the specific decay constant between treatments.

We observed a steady decay in the number of culturable isolates over the first 6 weeks of the study (Fig 1A). After 7 weeks, colonies were no longer detected in soil with surface-applied EcO157 (Fig 1A). For all other treatments, EcO157 were still detectable 8 weeks after the initial inoculation (Fig 1A). This falls within the range of *E*. *coli* detection durations reported in studies using a similar initial inoculation density (ca. 1 x 10^7^ cfu per gram dry weight of soil) and unautoclaved, manure-amended soil. For instance, previous work reported that this same strain was detectable between 32 and 113 days after inoculation, depending on the soil type used [14].

There was no significant difference in the weekly percent water loss between treatments, weeks, or the treatment-week interaction (ANOVA, all *p* > 0.05). This indicates that desiccation occurred at a consistent rate across all treatments, and over the course of the experiment.

A specific decay rate was calculated for each survival curve using the specific growth rate equation for bacteria (Eq 1; S1 Table) [30]. Overall, the surface-applied treatments decayed faster (had a more negative decay rate) than the incorporated treatments. The decay rates for the soil surface-applied treatment were significantly more negative than the decay rates for both manure treatments (Tukey’s HSD, *p* < 0.05; Fig 1B), indicating that manure attenuated the die-off. Manure provides important nutrients to *E*. *coli* that can prolong survival. In a study of *E*. *coli* O157 survival in 36 different soil types, dissolved organic C per unit of soil biomass was the soil property that best predicted *E*. *coli* survival [13]. Dissolved organic N also positively correlated with *E*. *coli* survival [13]. In addition to these nutritional protective effects, it is also known that *E*. *coli* survive longer when manure is injected into the soil, where cells are protected from UV exposure and drying [18].

### Phenotypic characterization of isolates

**Fig 2:**
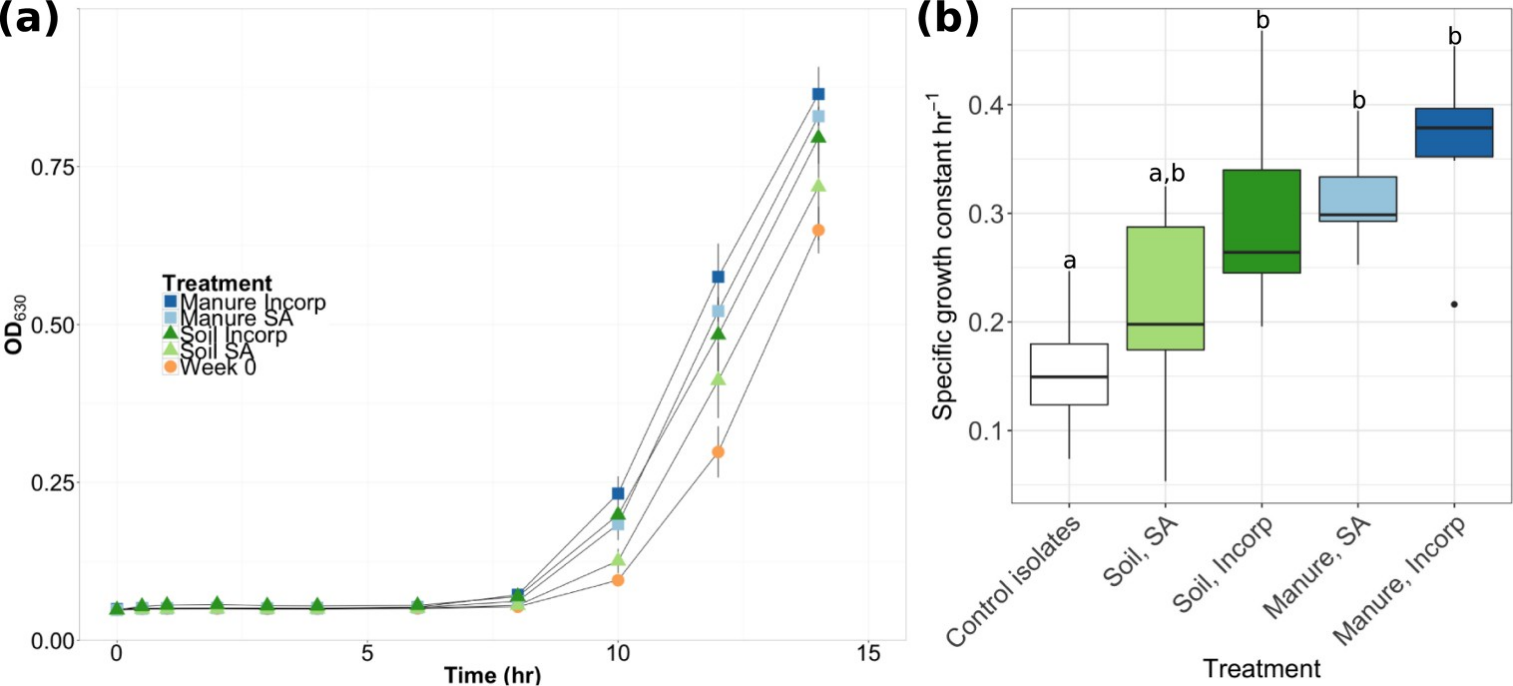
(a) Growth curves and (b) specific growth constants when leaving the lag phase (6 to 10 hrs) of *E*. *coli* O157:H7 EDL933 isolates in LB at 37°C, collected from each treatment type at week 6 and initial population isolates (week 0). Data represents an average (+/- 1SD) of six replicates. Different letters represent significant differences (*p* < 0.05) in the specific growth constant between treatments.

The specific growth rate for EcO157 reisolated from soil and grown in LB broth at 37°C differed significantly between treatments during the period where the isolates were leaving the lag phase (6 to 10 hrs) (Fig 2). Reisolates from all treatments except soil surface-applied left the lag phase at a significantly greater rate than the control (week 0) treatment (Fig 2B). A similar trend was observed when the specific growth rate was calculated for the full 14 hour period, but these differences were not significant (*p* > 0.05; S3 Fig). Growth was not detected at 15°C in LB. Similar growth patterns have been observed for naturalized *E*. *coli* populations in soils; while *E*. *coli* grew at or above 30°C, there was no growth at 15°C [39]. However, upon shifting microcosms from 15°C to 37°C, cell growth resumed. This suggests that *E*. *coli* can persist at 15°C and then return to growth once the conditions become suitable [39].

**Fig 3:**
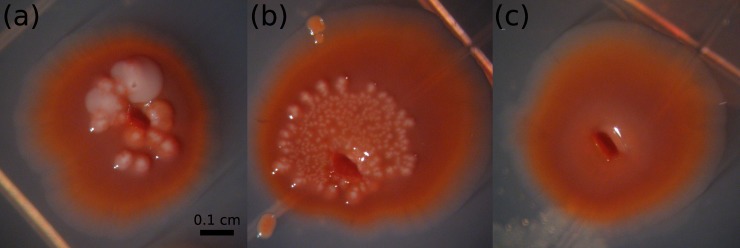
Sample morphologies of isolates grown on Congo Red agar for seven days at 30°C. Isolates (a) and (b) were collected from the surface-applied manure treatment after six weeks, and exemplify the red morphology overlain with raised white growth that was unique to isolates from this treatment. An isolate (c) was collected from the same soil microcosm after six weeks, and is representative of the smooth, uniform red morphology manifest by isolates collected from all treatments other than surface-applied manure. Pictures were taken under 16X magnification.

The reisolates from distinct treatments showed phenotypic differences when grown on CR agar for seven days at 30°C. Specifically, 7 out of 15 randomly selected week 6 isolates from the surface applied manure treatment developed a white growth on top of an otherwise red colony morphology (Fig 3). All other isolates, including the control (week 0) isolates, exhibited red colony morphologies. Red colonies indicate the production of curli, while white colonies indicate the absence of curli [28]. A hypothesis test for equal proportions indicated the colonies reisolated in week 6 from the surface applied manure had a significantly different proportion of red colonies on CR agar compared to the other treatments and times (p < 0.001). These CR morphology results suggest that prolonged exposure to different manure application methods, specifically to surface-applied manure in this case, resulted in distinct phenotypic populations in the soil microcosms. In unpublished data from an earlier study (S2 Fig), we found a similar trend. Namely, the ratio of isolates with a mixed red and white morphotype to isolates with a red morphotype was highest in the *E*. *coli* population sampled from the top 2 cm of soil in a column treated with surface-applied manure. Additionally, in a study investigating curli expression in nine different *E*. *coli* O157:H7 strains over three repetitive soil incubations lasting 5–6 days, the proportion of isolates expressing curli consistently decreased [23].

### Genome resequencing

Given the observed differences in growth rate and CR morphology, we undertook whole-genome resequencing in order to understand the genetic underpinnings for the observed phenotypic differences. Surprisingly, our analyses did not identify any consistent mutations that correlated with either of the observed phenotypic differences. In fact, we were only able to identify single mutations in two of the 94 strains we sampled when compared to the parental strain [24]. Both mutations were missense, and present in only one of the reisolates each (Table 2).

**Table 2:** Single nucleotide polymorphisms (SNPs) identified in resequenced isolates of *E*. *coli* O157:H7 EDL933 Δ*stx*_1-2_.

Nucleotide position*	Feature	GenBank accession number	Mutation type	Wild type	Mutant	Strain	Strain description
380467	AidA-I adhesion-like protein	AIG66568.1	Missense	A	G	SWL0019	Week 3, surface-applied, with manure
1380750	Hypothetical protein	AIG67612.1	Missense	C	T	SWL0050	Week 5, incorporated, soil only

*Positions are relative to *E*. *coli* O157:H7 EDL933 (GenBank accession no. CP008957.1) [24].

The reisolate from week 5 encoded a SNP in a gene encoding a hypothetical protein (GenBank accession no. AIG66568.1) and the one from week 3 harbored a SNP in *aidA-*I, encoding an adhesion-like protein (GenBank accession no. AIG67612.1) [24]. The latter was confirmed with Sanger sequencing. A summary table of the sequencing reads mapped for each isolate in CLC Workbench is provided in S2 Table. AidA-I was first identified as conferring the diffuse adherence phenotype on enteropathogenic *E*. *coli* strains [40,41], characterized by uniform adherence to the whole host cell surface [42]. It also enhances bacterial aggregation, biofilm formation, and invasion of host cells [43,44]. While the occurrence of one mutation of this nature in the 96 reisolates examined is not evidence for a significant pattern, it is of interest given the idiosyncratic nature of food-related illnesses and the observation that far more individuals are exposed than become ill.

No other systematic differences were detected in the genomes of any of the other isolates. There was no significant difference between the number of gaps in any gene that could be explained by week or treatment (*p* > 0.05; S3 Table). Gaps did tend to occur in the same genes across isolates, including week 0 isolates, suggesting that although our initial population differed from the reference sequence, these differences were unrelated to treatment (S3 Table). Furthermore, there was no significant effect of treatment or time on the number of unmapped reads that could be annotated as predicted features or assigned to any specific taxa, or subsystem via MGRAST (S4 Fig; S4 Table).

Combined, the SNP, gap, and discarded read results demonstrate that there were no systematic genomic differences that can be explained by week or manure application treatment. In other words, 92 of the 94 reisolates were genetically identical to the parent strain, while two isolates contained one SNP each. This suggests that survival was not driven by genetic selection at the individual level. However, the clear trend seen in the CR phenotypes between treatments leads us to conclude that individual survival was not completely stochastic. No genetic changes were detected in any known curli regulators in any of the reisolates we resequenced (S5 Table). Therefore, the mechanism behind differential expression resulting in the altered phenotypes remains unknown. One possibility for the observed phenotypic differences is epigenetic regulation of the curli genes; DNA methylation has been shown to affect curli expression in *Salmonella* [45]. Therefore, methylation may be an important epigenetic control on curli production in *E*. *coli* as well, although further work will be required to assess its impact in this strain.

Finally, our results lead us to hypothesize that the phenotypic differences we previously observed between populations of *E*. *coli* transported from soil were driven by population-level selection dependent on some combination of the environment (incorporated manure versus surface-applied manure) and the transport process, not on the adaptation of one strain to those conditions [21]. It has been shown that pH, water content, and percent organic matter can partially account for genotype variation across a landscape for microbial species including *E*. *coli* [46–48], and that the variability in survival seen amongst *E*. *coli* strains is as great as the variability in survival caused by soil matrix characteristics for a single *E*. *coli* strain [7,13]. In light of those findings and the present work, it seems essential that risk assessments aimed at understanding the persistence of *E*. *coli* in soil should be based on the survival of multiple strains; although this does not always occur in practice [49]. Further work could seek to test (1) if differing manure management practices influence the population-level genetic structure of *E*. *coli*, (2) whether and how different soil landscapes interact with manure management practices to influence the population-level genetic structure of *E*. *coli* and (3) whether the transport process itself is another variable that selects for distinct *E*. *coli* populations sourced from subsurface drainage. This knowledge would help guide manure management practices to avoid creation of reservoirs of stress-tolerance genes, such as those promoting curli formation, in both the soil and surface-water landscapes.

## Conclusions

Through whole-genome sequencing, we demonstrated that there was no systematic selective pressure on genomes of pathogenic *E*. *coli* O157:H7 EDL933 Δ*stx*_1-2_ (EcO157) exposed to cyclic drying in the soil and different manure application methods. This result suggests that more understanding is needed about the impacts of how manure application method affects population-wide phenotypic patterns previously observed in *E*. *coli* isolated from the soil. EcO157 survival in this experiment was not completely stochastic, as evinced by both differing curli phenotypes and specific growth rates by manure application method. We suggest that future research investigate whether and how population-level genetic selection results from different manure application methods. This could provide a much-needed mechanistic basis for choosing manure management techniques that minimize the persistence of pathogenic *E*. *coli* in the agricultural environment.

## Supporting information

S1 Table Specific decay rate for EcO157 in each microcosm. Calculated according to Neidhardt et al. (1990). The coefficient of determination (r^2^) is shown for goodness of fit.
(DOCX)

S2 Table Metrics from mapping reads to reference genome in CLC Workbench. The reference genome (main chromosome plus plasmid, GenBank accession nos. CP008957.1 and CP008958.1) is 5,639,239 bp [24].
(DOCX)

S3 Table Comparison of number of gaps found in genes in weeks 0, 3, 5/6, and 8 isolates. Using a Poisson model for count data, and week-treatment as the explanatory variable. Protein IDs are the GenBank accession numbers for the proteins from the chromosome and plasmid of *E*. *coli* O157:H7 str. EDL933 (GenBank accession nos. CP008957.1 and CP008958.1) [24].
(DOCX)

S4 Table Significance of annotated reads as a linear function of week-treatment. The MG-RAST predicted features used to annotate reads were: the top 25 genuses (by frequency) and functional subsystems, except for those that were correlated with all other functional subsystems. Subsequently, all *p* values were then ordered and compared to threshold values calculated using the Benjamini-Hochberg (BH) procedure, to account for multiple comparisons. The false discovery rate (**α**) was set at 0.25 to ensure no possible significant factors were overlooked. A *p* value less than the corresponding threshold is considered significant.
(DOCX)

S5 Table Summary of known curli regulators [20]. The location of each regulator in the *E*. *coli* O157:H7 EDL933 reference genome used in this study is provided (GenBank accession no. CP008957.1) [24], along with an indication of whether a gap or SNP was found in the regulator through mapping to the reference genome on CLC Workbench.
(DOCX)

S1 Fig Schematic of bioinformatics procedure. (PDF)

S2 Fig Previously unpublished Congo Red (CR) morphotype data collected by Truhlar et al. (2015). The ratio of isolates with a mixed red and white morphotype to isolates with a red morphotype was highest in the population sampled from the top 2 cm of soil in the surface applied treatment column. A red colony morphotype on CR agar indicates curli production, while a white morphotype indicates no curli production. For all treatments, the red morphotype was the predominant morphotype in the population. Control treatments had no added manure, incorporated treatments mixed the manure into the top 5 cm of soil, and surface-applied treatments left the manure on the soil surface. The manure sample was taken from the manure used in all treatments.
(PDF)

S3 Fig Specific growth constants for *E*. *coli* O157:H7 EDL933 isolates. Collected from each treatment type at week 6, and the control (week 0). Grown over a 14-hr period in LB broth at 37°C. Data represents six replicates for each treatment.
(PDF)

S4 Fig Discarded reads annotated with a predicted feature by the MG-RAST server. The reads are plotted by both the week the isolate was collected and the soil-manure treatment to which the isolate was exposed. Four points with values greater than 25,000 were excluded to make the plot easier to read. These points came from week 3 soil-incorporated, week 5/6 manure-incorporated, week 5/6 manure surface-applied, and week 8 manure-incorporated strains.
(PDF)

S1 Dataset Soil temperature at eight-inch depth for April-October 2009–2017. Downloaded on September 14, 2018 from the National Resources Conservation Service, Soil Climate Analysis Network (SCAN), for the Geneva, NY station.
(CSV)

S2 Dataset Microcosm weights over the eight weeks of the experiment. Weights were taken prior to and following sampling each week. This dataset also contains the weight of water added each week prior to sampling to return the microcosm to 60% water holding capacity, and the weekly percent water loss. The latter was calculated as the weight of water added to return to pre-desiccation weight, divided by the pre-desiccation water weight, assuming 60% WHC.
(CSV)
