# Supplementary material for: Absence of genetic selection in a pathogenic Escherichia coli strain exposed to the manure-amended soil environment
Source: PLoS One. 2018 Dec 7;13(12):e0208346. doi: 10.1371/journal.pone.0208346 (PMC6286177; doi:10.1371/journal.pone.0208346)
Supplement: S1 Table — Calculated according to Neidhardt et al. (1990). The coefficient of determination (r2) is shown for goodness of fit. (DOCX) [file pone.0208346.s001.docx]

| **Treatment** | **Pot** | **Decay rate** | **r^2^** |
| --- | --- | --- | --- |
| InocSoil_SA | 7 | -0.14 | 0.96 |
| InocSoil_SA | 8 | -0.12 | 0.96 |
| InocSoil_SA | 21 | -0.10 | 0.90 |
| InocSoil_SA | 22 | -0.10 | 0.81 |
| InocSoil_Incorp | 9 | -0.08 | 0.89 |
| InocSoil_Incorp | 10 | -0.06 | 0.95 |
| InocSoil_Incorp | 23 | -0.08 | 0.91 |
| InocSoil_Incorp | 24 | -0.10 | 0.89 |
| InocManure_SA | 11 | -0.08 | 0.81 |
| InocManure_SA | 12 | -0.12 | 0.92 |
| InocManure_SA | 25 | -0.10 | 0.84 |
| InocManure_SA | 26 | -0.09 | 0.80 |
| InocManure_Incorp | 13 | -0.06 | 0.84 |
| InocManure_Incorp | 14 | -0.07 | 0.88 |
| InocManure_Incorp | 27 | -0.08 | 0.93 |
| InocManure_Incorp | 28 | -0.09 | 0.91 |
