# Supplementary material for: Absence of genetic selection in a pathogenic Escherichia coli strain exposed to the manure-amended soil environment
Source: PLoS One. 2018 Dec 7;13(12):e0208346. doi: 10.1371/journal.pone.0208346 (PMC6286177; doi:10.1371/journal.pone.0208346)
Supplement: S2 Table — The reference genome (main chromosome plus plasmid, GenBank accession nos. CP008957.1 and CP008958.1) is 5,639,239 bp [24]. (DOCX) [file pone.0208346.s002.docx]

| **Isolate** | **Total reads** | **Mapped reads** | **Unmapped reads** | **Percentage unmapped reads** | **Genome-wide average coverage** | **Number of bp with zero coverage** | **Percentage of genome with zero coverage** |
| --- | --- | --- | --- | --- | --- | --- | --- |
| SWL0001 | 5115512 | 5008033 | 107479 | 2.1% | 110 | 832 | 0.015% |
| SWL0002 | 3463972 | 3379949 | 84023 | 2.5% | 76 | 987 | 0.018% |
| SWL0003 | 2404902 | 2335309 | 69593 | 3.0% | 53 | 1376 | 0.024% |
| SWL0004 | 2441250 | 2304023 | 137227 | 6.0% | 52 | 1066 | 0.019% |
| SWL0005 | 3251463 | 3159144 | 92319 | 2.9% | 70 | 1127 | 0.020% |
| SWL0006 | 3276316 | 3209806 | 66510 | 2.1% | 73 | 1250 | 0.022% |
| SWL0007 | 4451822 | 4334152 | 117670 | 2.7% | 97 | 926 | 0.016% |
| SWL0008 | 3713149 | 3621714 | 91435 | 2.5% | 82 | 986 | 0.017% |
| SWL0009 | 4142715 | 4044635 | 98080 | 2.4% | 89 | 971 | 0.017% |
| SWL0010 | 2427728 | 2333294 | 94434 | 4.0% | 52 | 1111 | 0.020% |
| SWL0012 | 3536595 | 3458660 | 77935 | 2.3% | 77 | 1075 | 0.019% |
| SWL0013 | 3614221 | 3497951 | 116270 | 3.3% | 79 | 1051 | 0.019% |
| SWL0014 | 2758139 | 2667328 | 90811 | 3.4% | 59 | 1122 | 0.020% |
| SWL0015 | 2204881 | 2091796 | 113085 | 5.4% | 48 | 1286 | 0.023% |
| SWL0016 | 3628416 | 3549309 | 79107 | 2.2% | 79 | 1645 | 0.029% |
| SWL0017 | 3452624 | 3400978 | 51646 | 1.5% | 77 | 1052 | 0.019% |
| SWL0018 | 2919246 | 2837237 | 82009 | 2.9% | 64 | 1075 | 0.019% |
| SWL0019 | 2384764 | 2284697 | 100067 | 4.4% | 51 | 1268 | 0.022% |
| SWL0020 | 2275215 | 2066457 | 208758 | 10.1% | 48 | 1485 | 0.026% |
| SWL0021 | 3548739 | 3471336 | 77403 | 2.2% | 78 | 909 | 0.016% |
| SWL0022 | 2455883 | 2344974 | 110909 | 4.7% | 54 | 1159 | 0.021% |
| SWL0023 | 2767158 | 2607100 | 160058 | 6.1% | 57 | 1206 | 0.021% |
| SWL0024 | 1994310 | 1765660 | 228650 | 12.9% | 40 | 1376 | 0.024% |
| SWL0025 | 2202961 | 2058912 | 144049 | 7.0% | 47 | 1346 | 0.024% |
| SWL0026 | 3112547 | 2987157 | 125390 | 4.2% | 68 | 1011 | 0.018% |
| SWL0027 | 4331064 | 4245170 | 85894 | 2.0% | 95 | 947 | 0.017% |
| SWL0028 | 2860889 | 2697001 | 163888 | 6.1% | 61 | 1265 | 0.022% |
| SWL0029 | 3670006 | 3612451 | 57555 | 1.6% | 80 | 1112 | 0.020% |
| SWL0030 | 1814541 | 1769883 | 44658 | 2.5% | 41 | 1681 | 0.030% |
| SWL0031 | 2730522 | 2606682 | 123840 | 4.8% | 59 | 1276 | 0.023% |
| SWL0032 | 2324018 | 2268683 | 55335 | 2.4% | 52 | 1221 | 0.022% |
| SWL0033 | 2663637 | 2525443 | 138194 | 5.5% | 58 | 1224 | 0.022% |
| SWL0034 | 2533253 | 2450801 | 82452 | 3.4% | 56 | 1338 | 0.024% |
| SWL0035 | 5473772 | 5317958 | 155814 | 2.9% | 116 | 840 | 0.015% |
| SWL0036 | 1266599 | 1207174 | 59425 | 4.9% | 28 | 1460 | 0.026% |
| SWL0037 | 3468920 | 3397718 | 71202 | 2.1% | 75 | 970 | 0.017% |
| SWL0038 | 2894467 | 2746831 | 147636 | 5.4% | 62 | 1163 | 0.021% |
| SWL0039 | 3638577 | 3551097 | 87480 | 2.5% | 80 | 1020 | 0.018% |
| SWL0040 | 3778062 | 3709085 | 68977 | 1.9% | 84 | 1116 | 0.020% |
| SWL0041 | 3562279 | 3484006 | 78273 | 2.2% | 79 | 1020 | 0.018% |
| SWL0042 | 3459679 | 3329811 | 129868 | 3.9% | 75 | 953 | 0.017% |
| SWL0043 | 3202458 | 3083829 | 118629 | 3.8% | 70 | 1126 | 0.020% |
| SWL0044 | 4013766 | 3944224 | 69542 | 1.8% | 88 | 892 | 0.016% |
| SWL0045 | 4373289 | 4283928 | 89361 | 2.1% | 94 | 912 | 0.016% |
| SWL0046 | 3458706 | 3397807 | 60899 | 1.8% | 77 | 979 | 0.017% |
| SWL0047 | 2946169 | 2873500 | 72669 | 2.5% | 63 | 1260 | 0.022% |
| SWL0048 | 2613328 | 2552320 | 61008 | 2.4% | 57 | 1247 | 0.022% |
| SWL0049 | 1938180 | 1842619 | 95561 | 5.2% | 43 | 1346 | 0.024% |
| SWL0050 | 3510426 | 3418640 | 91786 | 2.7% | 77 | 1062 | 0.019% |
| SWL0051 | 3110401 | 2955209 | 155192 | 5.3% | 67 | 1061 | 0.019% |
| SWL0052 | 2599003 | 2517452 | 81551 | 3.2% | 57 | 1011 | 0.018% |
| SWL0053 | 3033119 | 2972523 | 60596 | 2.0% | 66 | 1209 | 0.021% |
| SWL0054 | 1875493 | 1748583 | 126910 | 7.3% | 39 | 1350 | 0.024% |
| SWL0055 | 2240281 | 2136059 | 104222 | 4.9% | 48 | 1324 | 0.023% |
| SWL0056 | 2620681 | 2507293 | 113388 | 4.5% | 57 | 1080 | 0.019% |
| SWL0057 | 3088347 | 3008942 | 79405 | 2.6% | 68 | 1120 | 0.020% |
| SWL0058 | 3029425 | 2838507 | 190918 | 6.7% | 63 | 1115 | 0.020% |
| SWL0059 | 2148067 | 2053371 | 94696 | 4.6% | 47 | 1330 | 0.024% |
| SWL0060 | 3211254 | 3063443 | 147811 | 4.8% | 69 | 1088 | 0.019% |
| SWL0061 | 3278275 | 3007683 | 270592 | 9.0% | 66 | 1077 | 0.019% |
| SWL0062 | 3710794 | 3607623 | 103171 | 2.9% | 80 | 886 | 0.016% |
| SWL0063 | 4163842 | 4061398 | 102444 | 2.5% | 90 | 940 | 0.017% |
| SWL0064 | 3361621 | 3275006 | 86615 | 2.6% | 73 | 1164 | 0.021% |
| SWL0065 | 2489341 | 2363361 | 125980 | 5.3% | 54 | 1271 | 0.023% |
| SWL0066 | 3947989 | 3883124 | 64865 | 1.7% | 87 | 1045 | 0.019% |
| SWL0067 | 3611196 | 3496159 | 115037 | 3.3% | 78 | 1026 | 0.018% |
| SWL0068 | 3056001 | 2852790 | 203211 | 7.1% | 66 | 1212 | 0.021% |
| SWL0069 | 3232967 | 3148094 | 84873 | 2.7% | 71 | 1056 | 0.019% |
| SWL0070 | 3135195 | 3016236 | 118959 | 3.9% | 68 | 1102 | 0.020% |
| SWL0071 | 1872896 | 1738028 | 134868 | 7.8% | 40 | 1452 | 0.026% |
| SWL0072 | 2746907 | 2671809 | 75098 | 2.8% | 61 | 1263 | 0.022% |
| SWL0073 | 3039790 | 2779227 | 260563 | 9.4% | 64 | 1066 | 0.019% |
| SWL0074 | 2205149 | 2068738 | 136411 | 6.6% | 47 | 1320 | 0.023% |
| SWL0075 | 3297976 | 3202865 | 95111 | 3.0% | 72 | 1134 | 0.020% |
| SWL0076 | 5278034 | 5171966 | 106068 | 2.1% | 112 | 819 | 0.015% |
| SWL0077 | 3887568 | 3800661 | 86907 | 2.3% | 86 | 1178 | 0.021% |
| SWL0078 | 1940277 | 1825813 | 114464 | 6.3% | 41 | 1384 | 0.025% |
| SWL0079 | 2938780 | 2845342 | 93438 | 3.3% | 63 | 1078 | 0.019% |
| SWL0080 | 3386280 | 3321620 | 64660 | 1.9% | 75 | 1309 | 0.023% |
| SWL0081 | 2437111 | 2338500 | 98611 | 4.2% | 53 | 1310 | 0.023% |
| SWL0082 | 3822114 | 3760757 | 61357 | 1.6% | 83 | 1048 | 0.019% |
| SWL0083 | 3753978 | 3613259 | 140719 | 3.9% | 81 | 1052 | 0.019% |
| SWL0084 | 3402067 | 3336196 | 65871 | 2.0% | 74 | 992 | 0.018% |
| SWL0085 | 3173600 | 3104967 | 68633 | 2.2% | 71 | 1123 | 0.020% |
| SWL0086 | 3110182 | 2833383 | 276799 | 9.8% | 65 | 1308 | 0.023% |
| SWL0087 | 3710315 | 3510998 | 199317 | 5.7% | 79 | 1030 | 0.018% |
| SWL0088 | 4427458 | 4076768 | 350690 | 8.6% | 91 | 1015 | 0.018% |
| SWL0089 | 2711828 | 2621168 | 90660 | 3.5% | 60 | 1048 | 0.019% |
| SWL0090 | 2998881 | 2919782 | 79099 | 2.7% | 67 | 1309 | 0.023% |
| SWL0091 | 3026168 | 2937974 | 88194 | 3.0% | 65 | 1138 | 0.020% |
| SWL0092 | 3204218 | 3113797 | 90421 | 2.9% | 71 | 1094 | 0.019% |
| SWL0093 | 2709041 | 2661366 | 47675 | 1.8% | 60 | 1248 | 0.022% |
| SWL0094 | 3278896 | 3167775 | 111121 | 3.5% | 72 | 1088 | 0.019% |
| SWL0095 | 4369886 | 4254489 | 115397 | 2.7% | 94 | 895 | 0.016% |
