# Supplementary material for: Absence of genetic selection in a pathogenic Escherichia coli strain exposed to the manure-amended soil environment
Source: PLoS One. 2018 Dec 7;13(12):e0208346. doi: 10.1371/journal.pone.0208346 (PMC6286177; doi:10.1371/journal.pone.0208346)
Supplement: S3 Table — Using a Poisson model for count data, and week-treatment as the explanatory variable. Protein IDs are the GenBank accession numbers for the proteins from the chromosome and plasmid of E. coli O157:H7 str. EDL933 (GenBank accession nos. CP008957.1 and CP008958.1) [24]. (DOCX) [file pone.0208346.s003.docx]

| **Annotation** | **Protein ID** | **Week-Treatment** | **Estimate** | **z value** | **Pr(>\|z\|)** |
| --- | --- | --- | --- | --- | --- |
| Hypothetical protein CDS | AIG66563.1 | WeekTreatWeek 0, Control | -2.2E+01 | -1.2E-03 | 1.00 |
|  |  | WeekTreatWeek 3, M, Inc | 8.0E-10 | 3.1E-14 | 1.00 |
|  |  | WeekTreatWeek 3, M, SA | 8.0E-10 | 3.1E-14 | 1.00 |
|  |  | WeekTreatWeek 3, S, Inc | 8.0E-10 | 3.1E-14 | 1.00 |
|  |  | WeekTreatWeek 3, S, SA | 8.0E-10 | 3.0E-14 | 1.00 |
|  |  | WeekTreatWeek 5/6, M, Inc | 8.0E-10 | 3.5E-14 | 1.00 |
|  |  | WeekTreatWeek 5/6, M, SA | 8.0E-10 | 3.5E-14 | 1.00 |
|  |  | WeekTreatWeek 5/6, S, Inc | 8.0E-10 | 3.5E-14 | 1.00 |
|  |  | WeekTreatWeek 5/6, S, SA | 2.1E+01 | 1.1E-03 | 1.00 |
|  |  | WeekTreatWeek 8, M, Inc | 8.0E-10 | 3.1E-14 | 1.00 |
|  |  | WeekTreatWeek 8, M, SA | 8.0E-10 | 3.1E-14 | 1.00 |
|  |  | WeekTreatWeek 8, S, Inc | 8.0E-10 | 3.1E-14 | 1.00 |
| Hypothetical protein CDS | AIG66691.1 | WeekTreatWeek 0, Control | -2.2E+01 | -1.2E-03 | 1.00 |
|  |  | WeekTreatWeek 3, M, Inc | 9.9E-10 | 3.9E-14 | 1.00 |
|  |  | WeekTreatWeek 3, M, SA | 9.9E-10 | 3.9E-14 | 1.00 |
|  |  | WeekTreatWeek 3, S, Inc | 9.9E-10 | 3.9E-14 | 1.00 |
|  |  | WeekTreatWeek 3, S, SA | 9.9E-10 | 3.7E-14 | 1.00 |
|  |  | WeekTreatWeek 5/6, M, Inc | 9.9E-10 | 4.4E-14 | 1.00 |
|  |  | WeekTreatWeek 5/6, M, SA | 9.9E-10 | 4.4E-14 | 1.00 |
|  |  | WeekTreatWeek 5/6, S, Inc | 2.0E+01 | 1.0E-03 | 1.00 |
|  |  | WeekTreatWeek 5/6, S, SA | 2.0E+01 | 1.0E-03 | 1.00 |
|  |  | WeekTreatWeek 8, M, Inc | 9.9E-10 | 3.9E-14 | 1.00 |
|  |  | WeekTreatWeek 8, M, SA | 9.9E-10 | 3.9E-14 | 1.00 |
|  |  | WeekTreatWeek 8, S, Inc | 9.9E-10 | 3.9E-14 | 1.00 |
| Type III restriction enzyme, res subunit:DEAD/DEAH box helicase, N- terminal CDS | AIG67225.1 | WeekTreatWeek 0, Control | -7.4E-17 | -1.7E-16 | 1.00 |
|  |  | WeekTreatWeek 3, M, Inc | 5.8E-17 | 9.5E-17 | 1.00 |
|  |  | WeekTreatWeek 3, M, SA | 7.2E-17 | 1.2E-16 | 1.00 |
|  |  | WeekTreatWeek 3, S, Inc | 9.6E-17 | 1.6E-16 | 1.00 |
|  |  | WeekTreatWeek 3, S, SA | 3.4E-17 | 5.3E-17 | 1.00 |
|  |  | WeekTreatWeek 5/6, M, Inc | -8.7E-02 | -1.6E-01 | 0.87 |
|  |  | WeekTreatWeek 5/6, M, SA | 4.5E-17 | 8.4E-17 | 1.00 |
|  |  | WeekTreatWeek 5/6, S, Inc | 8.0E-02 | 1.5E-01 | 0.88 |
|  |  | WeekTreatWeek 5/6, S, SA | -8.7E-02 | -1.6E-01 | 0.87 |
|  |  | WeekTreatWeek 8, M, Inc | 4.2E-17 | 7.0E-17 | 1.00 |
|  |  | WeekTreatWeek 8, M, SA | 4.0E-17 | 6.5E-17 | 1.00 |
|  |  | WeekTreatWeek 8, S, Inc | 4.9E-17 | 8.1E-17 | 1.00 |
| Transposase C CDS | AIG67229.1 | WeekTreatWeek 0, Control | -1.6E+00 | -1.6E+00 | 0.11 |
|  |  | WeekTreatWeek 3, M, Inc | 5.1E-01 | 4.2E-01 | 0.68 |
|  |  | WeekTreatWeek 3, M, SA | 1.2E+00 | 1.1E+00 | 0.28 |
|  |  | WeekTreatWeek 3, S, Inc | 5.1E-01 | 4.2E-01 | 0.68 |
|  |  | WeekTreatWeek 3, S, SA | 1.5E-14 | 1.1E-14 | 1.00 |
|  |  | WeekTreatWeek 5/6, M, Inc | 2.2E-01 | 1.9E-01 | 0.85 |
|  |  | WeekTreatWeek 5/6, M, SA | 1.2E+00 | 1.1E+00 | 0.26 |
|  |  | WeekTreatWeek 5/6, S, Inc | 2.2E-01 | 1.9E-01 | 0.85 |
|  |  | WeekTreatWeek 5/6, S, SA | 1.1E+00 | 1.0E+00 | 0.32 |
|  |  | WeekTreatWeek 8, M, Inc | 1.4E+00 | 1.3E+00 | 0.19 |
|  |  | WeekTreatWeek 8, M, SA | 5.1E-01 | 4.2E-01 | 0.68 |
|  |  | WeekTreatWeek 8, S, Inc | -1.8E-01 | -1.3E-01 | 0.90 |
| Transposase CDS | AIG67230.1 | WeekTreatWeek 0, Control | -9.2E-01 | -1.3E+00 | 0.20 |
|  |  | WeekTreatWeek 3, M, Inc | 9.2E-01 | 1.1E+00 | 0.26 |
|  |  | WeekTreatWeek 3, M, SA | 7.3E-01 | 8.8E-01 | 0.38 |
|  |  | WeekTreatWeek 3, S, Inc | 9.2E-01 | 1.1E+00 | 0.26 |
|  |  | WeekTreatWeek 3, S, SA | 6.9E-01 | 8.0E-01 | 0.42 |
|  |  | WeekTreatWeek 5/6, M, Inc | 2.2E-01 | 2.7E-01 | 0.78 |
|  |  | WeekTreatWeek 5/6, M, SA | 8.3E-01 | 1.1E+00 | 0.28 |
|  |  | WeekTreatWeek 5/6, S, Inc | 8.3E-01 | 1.1E+00 | 0.28 |
|  |  | WeekTreatWeek 5/6, S, SA | 7.3E-01 | 9.5E-01 | 0.34 |
|  |  | WeekTreatWeek 8, M, Inc | 9.2E-01 | 1.1E+00 | 0.26 |
|  |  | WeekTreatWeek 8, M, SA | 9.2E-01 | 1.1E+00 | 0.26 |
|  |  | WeekTreatWeek 8, S, Inc | 5.1E-01 | 5.9E-01 | 0.56 |
| Hypothetical protein CDS | AIG67231.1 | WeekTreatWeek 0, Control | -2.2E-01 | -4.5E-01 | 0.66 |
|  |  | WeekTreatWeek 3, M, Inc | 2.2E-01 | 3.5E-01 | 0.73 |
|  |  | WeekTreatWeek 3, M, SA | 2.2E-01 | 3.5E-01 | 0.73 |
|  |  | WeekTreatWeek 3, S, Inc | 2.2E-01 | 3.5E-01 | 0.73 |
|  |  | WeekTreatWeek 3, S, SA | -1.0E-15 | -1.5E-15 | 1.00 |
|  |  | WeekTreatWeek 5/6, M, Inc | -1.8E-01 | -3.0E-01 | 0.77 |
|  |  | WeekTreatWeek 5/6, M, SA | 1.4E-01 | 2.3E-01 | 0.82 |
|  |  | WeekTreatWeek 5/6, S, Inc | 1.4E-01 | 2.3E-01 | 0.82 |
|  |  | WeekTreatWeek 5/6, S, SA | -6.5E-02 | -1.1E-01 | 0.91 |
|  |  | WeekTreatWeek 8, M, Inc | 2.2E-01 | 3.5E-01 | 0.73 |
|  |  | WeekTreatWeek 8, M, SA | 4.1E-02 | 6.1E-02 | 0.95 |
|  |  | WeekTreatWeek 8, S, Inc | 2.2E-01 | 3.5E-01 | 0.73 |
| Antigen 43 precursor CDS | AIG67322.1 | WeekTreatWeek 0, Control | -2.1E+01 | -1.9E-03 | 1.00 |
|  |  | WeekTreatWeek 3, M, Inc | 2.0E+01 | 1.7E-03 | 1.00 |
|  |  | WeekTreatWeek 3, M, SA | -3.5E-10 | -2.3E-14 | 1.00 |
|  |  | WeekTreatWeek 3, S, Inc | -3.5E-10 | -2.3E-14 | 1.00 |
|  |  | WeekTreatWeek 3, S, SA | -3.5E-10 | -2.2E-14 | 1.00 |
|  |  | WeekTreatWeek 5/6, M, Inc | 1.9E+01 | 1.6E-03 | 1.00 |
|  |  | WeekTreatWeek 5/6, M, SA | -3.5E-10 | -2.6E-14 | 1.00 |
|  |  | WeekTreatWeek 5/6, S, Inc | 1.9E+01 | 1.6E-03 | 1.00 |
|  |  | WeekTreatWeek 5/6, S, SA | 1.9E+01 | 1.6E-03 | 1.00 |
|  |  | WeekTreatWeek 8, M, Inc | -3.5E-10 | -2.3E-14 | 1.00 |
|  |  | WeekTreatWeek 8, M, SA | -3.5E-10 | -2.3E-14 | 1.00 |
|  |  | WeekTreatWeek 8, S, Inc | -3.5E-10 | -2.3E-14 | 1.00 |
| Antigen 43 precursor CDS | AIG67323.1 | WeekTreatWeek 0, Control | 9.4E-18 | 2.1E-17 | 1.00 |
|  |  | WeekTreatWeek 3, M, Inc | 8.7E-17 | 1.4E-16 | 1.00 |
|  |  | WeekTreatWeek 3, M, SA | -2.0E-18 | -3.3E-18 | 1.00 |
|  |  | WeekTreatWeek 3, S, Inc | 9.0E-17 | 1.5E-16 | 1.00 |
|  |  | WeekTreatWeek 3, S, SA | -9.1E-17 | -1.4E-16 | 1.00 |
|  |  | WeekTreatWeek 5/6, M, Inc | 8.0E-02 | 1.5E-01 | 0.88 |
|  |  | WeekTreatWeek 5/6, M, SA | 1.5E-01 | 3.0E-01 | 0.77 |
|  |  | WeekTreatWeek 5/6, S, Inc | -8.7E-02 | -1.6E-01 | 0.87 |
|  |  | WeekTreatWeek 5/6, S, SA | 8.0E-02 | 1.5E-01 | 0.88 |
|  |  | WeekTreatWeek 8, M, Inc | 6.1E-17 | 1.0E-16 | 1.00 |
|  |  | WeekTreatWeek 8, M, SA | 4.4E-17 | 7.3E-17 | 1.00 |
|  |  | WeekTreatWeek 8, S, Inc | 6.5E-17 | 1.1E-16 | 1.00 |
| Ferredoxin reductase CDS | AIG67324.1 | WeekTreatWeek 0, Control | -2.2E-01 | -4.5E-01 | 0.66 |
|  |  | WeekTreatWeek 3, M, Inc | 2.2E-01 | 3.5E-01 | 0.73 |
|  |  | WeekTreatWeek 3, M, SA | -4.7E-01 | -6.2E-01 | 0.54 |
|  |  | WeekTreatWeek 3, S, Inc | -8.8E-01 | -1.0E+00 | 0.31 |
|  |  | WeekTreatWeek 3, S, SA | 2.2E-01 | 3.3E-01 | 0.74 |
|  |  | WeekTreatWeek 5/6, M, Inc | -6.5E-02 | -1.1E-01 | 0.91 |
|  |  | WeekTreatWeek 5/6, M, SA | -1.8E-01 | -3.0E-01 | 0.77 |
|  |  | WeekTreatWeek 5/6, S, Inc | -4.7E-01 | -7.3E-01 | 0.47 |
|  |  | WeekTreatWeek 5/6, S, SA | -1.8E-01 | -3.0E-01 | 0.77 |
|  |  | WeekTreatWeek 8, M, Inc | -1.8E-01 | -2.6E-01 | 0.80 |
|  |  | WeekTreatWeek 8, M, SA | -8.8E-01 | -1.0E+00 | 0.31 |
|  |  | WeekTreatWeek 8, S, Inc | -8.8E-01 | -1.0E+00 | 0.31 |
| Transposase CDS | AIG67682.1 | WeekTreatWeek 0, Control | -5.1E-01 | -8.8E-01 | 0.38 |
|  |  | WeekTreatWeek 3, M, Inc | -1.8E-01 | -2.2E-01 | 0.82 |
|  |  | WeekTreatWeek 3, M, SA | 5.1E-01 | 7.2E-01 | 0.47 |
|  |  | WeekTreatWeek 3, S, Inc | -1.8E-01 | -2.2E-01 | 0.82 |
|  |  | WeekTreatWeek 3, S, SA | -4.1E-01 | -4.4E-01 | 0.66 |
|  |  | WeekTreatWeek 5/6, M, Inc | -1.8E-01 | -2.6E-01 | 0.80 |
|  |  | WeekTreatWeek 5/6, M, SA | 1.1E-01 | 1.6E-01 | 0.88 |
|  |  | WeekTreatWeek 5/6, S, Inc | 2.2E-01 | 3.3E-01 | 0.74 |
|  |  | WeekTreatWeek 5/6, S, SA | -2.8E-02 | -4.1E-02 | 0.97 |
|  |  | WeekTreatWeek 8, M, Inc | -1.8E-01 | -2.2E-01 | 0.82 |
|  |  | WeekTreatWeek 8, M, SA | -1.8E-01 | -2.2E-01 | 0.82 |
|  |  | WeekTreatWeek 8, S, Inc | -1.8E-01 | -2.2E-01 | 0.82 |
| Antigen 43 precursor CDS | AIG67774.1 | WeekTreatWeek 0, Control | -1.6E+00 | -1.6E+00 | 0.11 |
|  |  | WeekTreatWeek 3, M, Inc | -2.0E+01 | -1.9E-03 | 1.00 |
|  |  | WeekTreatWeek 3, M, SA | -2.0E+01 | -1.9E-03 | 1.00 |
|  |  | WeekTreatWeek 3, S, Inc | 5.1E-01 | 4.2E-01 | 0.68 |
|  |  | WeekTreatWeek 3, S, SA | -1.3E-14 | -8.9E-15 | 1.00 |
|  |  | WeekTreatWeek 5/6, M, Inc | -2.0E+01 | -2.7E-03 | 1.00 |
|  |  | WeekTreatWeek 5/6, M, SA | -2.0E+01 | -2.7E-03 | 1.00 |
|  |  | WeekTreatWeek 5/6, S, Inc | -2.0E+01 | -2.7E-03 | 1.00 |
|  |  | WeekTreatWeek 5/6, S, SA | -1.8E-01 | -1.5E-01 | 0.88 |
|  |  | WeekTreatWeek 8, M, Inc | -2.0E+01 | -1.9E-03 | 1.00 |
|  |  | WeekTreatWeek 8, M, SA | -2.0E+01 | -1.9E-03 | 1.00 |
|  |  | WeekTreatWeek 8, S, Inc | -2.0E+01 | -1.9E-03 | 1.00 |
| Putative vimentin CDS | AIG67776.1 | WeekTreatWeek 0, Control | 2.2E-17 | 4.8E-17 | 1.00 |
|  |  | WeekTreatWeek 3, M, Inc | -1.3E-17 | -2.2E-17 | 1.00 |
|  |  | WeekTreatWeek 3, M, SA | -1.3E-17 | -2.2E-17 | 1.00 |
|  |  | WeekTreatWeek 3, S, Inc | -1.3E-17 | -2.2E-17 | 1.00 |
|  |  | WeekTreatWeek 3, S, SA | -1.3E-17 | -2.1E-17 | 1.00 |
|  |  | WeekTreatWeek 5/6, M, Inc | -1.3E-17 | -2.5E-17 | 1.00 |
|  |  | WeekTreatWeek 5/6, M, SA | -1.3E-17 | -2.5E-17 | 1.00 |
|  |  | WeekTreatWeek 5/6, S, Inc | -1.3E-17 | -2.5E-17 | 1.00 |
|  |  | WeekTreatWeek 5/6, S, SA | -1.3E-17 | -2.5E-17 | 1.00 |
|  |  | WeekTreatWeek 8, M, Inc | -1.3E-17 | -2.2E-17 | 1.00 |
|  |  | WeekTreatWeek 8, M, SA | -1.3E-17 | -2.2E-17 | 1.00 |
|  |  | WeekTreatWeek 8, S, Inc | -1.3E-17 | -2.2E-17 | 1.00 |
| Hypothetical protein CDS | AIG68237.1 | WeekTreatWeek 0, Control | -2.3E+01 | -7.5E-04 | 1.00 |
|  |  | WeekTreatWeek 3, M, Inc | -2.5E-09 | -6.0E-14 | 1.00 |
|  |  | WeekTreatWeek 3, M, SA | -2.5E-09 | -6.0E-14 | 1.00 |
|  |  | WeekTreatWeek 3, S, Inc | 2.2E+01 | 6.9E-04 | 1.00 |
|  |  | WeekTreatWeek 3, S, SA | -2.5E-09 | -5.8E-14 | 1.00 |
|  |  | WeekTreatWeek 5/6, M, Inc | -2.5E-09 | -6.9E-14 | 1.00 |
|  |  | WeekTreatWeek 5/6, M, SA | -2.5E-09 | -6.8E-14 | 1.00 |
|  |  | WeekTreatWeek 5/6, S, Inc | -2.5E-09 | -6.8E-14 | 1.00 |
|  |  | WeekTreatWeek 5/6, S, SA | -2.5E-09 | -6.9E-14 | 1.00 |
|  |  | WeekTreatWeek 8, M, Inc | -2.5E-09 | -6.0E-14 | 1.00 |
|  |  | WeekTreatWeek 8, M, SA | -2.5E-09 | -6.0E-14 | 1.00 |
|  |  | WeekTreatWeek 8, S, Inc | -2.6E-09 | -6.1E-14 | 1.00 |
| Hypothetical protein CDS | AIG68238.1 | WeekTreatWeek 0, Control | -2.2E+01 | -1.2E-03 | 1.00 |
|  |  | WeekTreatWeek 3, M, Inc | -3.0E-09 | -1.2E-13 | 1.00 |
|  |  | WeekTreatWeek 3, M, SA | -3.0E-09 | -1.2E-13 | 1.00 |
|  |  | WeekTreatWeek 3, S, Inc | 2.2E+01 | 1.1E-03 | 1.00 |
|  |  | WeekTreatWeek 3, S, SA | -3.0E-09 | -1.1E-13 | 1.00 |
|  |  | WeekTreatWeek 5/6, M, Inc | -3.0E-09 | -1.4E-13 | 1.00 |
|  |  | WeekTreatWeek 5/6, M, SA | -3.0E-09 | -1.4E-13 | 1.00 |
|  |  | WeekTreatWeek 5/6, S, Inc | -3.0E-09 | -1.4E-13 | 1.00 |
|  |  | WeekTreatWeek 5/6, S, SA | -3.0E-09 | -1.4E-13 | 1.00 |
|  |  | WeekTreatWeek 8, M, Inc | -3.0E-09 | -1.2E-13 | 1.00 |
|  |  | WeekTreatWeek 8, M, SA | -3.0E-09 | -1.2E-13 | 1.00 |
|  |  | WeekTreatWeek 8, S, Inc | -3.0E-09 | -1.2E-13 | 1.00 |
| Phage capsid and scaffold protein CDS | AIG68254.1 | WeekTreatWeek 0, Control | -5.1E-01 | -8.8E-01 | 0.38 |
|  |  | WeekTreatWeek 3, M, Inc | 3.3E-01 | 4.5E-01 | 0.65 |
|  |  | WeekTreatWeek 3, M, SA | 5.1E-01 | 7.2E-01 | 0.47 |
|  |  | WeekTreatWeek 3, S, Inc | 3.3E-01 | 4.5E-01 | 0.65 |
|  |  | WeekTreatWeek 3, S, SA | 2.9E-01 | 3.8E-01 | 0.71 |
|  |  | WeekTreatWeek 5/6, M, Inc | 2.2E-01 | 3.3E-01 | 0.74 |
|  |  | WeekTreatWeek 5/6, M, SA | 3.3E-01 | 5.0E-01 | 0.62 |
|  |  | WeekTreatWeek 5/6, S, Inc | 3.3E-01 | 5.0E-01 | 0.62 |
|  |  | WeekTreatWeek 5/6, S, SA | 4.2E-01 | 6.5E-01 | 0.52 |
|  |  | WeekTreatWeek 8, M, Inc | 3.3E-01 | 4.5E-01 | 0.65 |
|  |  | WeekTreatWeek 8, M, SA | 1.1E-01 | 1.4E-01 | 0.89 |
|  |  | WeekTreatWeek 8, S, Inc | 3.3E-01 | 4.5E-01 | 0.65 |
| Phage capsid and scaffold protein CDS | AIG68258.1 | WeekTreatWeek 0, Control | -9.2E-01 | -1.3E+00 | 0.20 |
|  |  | WeekTreatWeek 3, M, Inc | -8.8E-01 | -7.1E-01 | 0.47 |
|  |  | WeekTreatWeek 3, M, SA | -1.7E+01 | -7.4E-03 | 0.99 |
|  |  | WeekTreatWeek 3, S, Inc | -8.8E-01 | -7.1E-01 | 0.47 |
|  |  | WeekTreatWeek 3, S, SA | -6.9E-01 | -5.7E-01 | 0.57 |
|  |  | WeekTreatWeek 5/6, M, Inc | -8.8E-01 | -8.8E-01 | 0.38 |
|  |  | WeekTreatWeek 5/6, M, SA | -8.8E-01 | -8.8E-01 | 0.38 |
|  |  | WeekTreatWeek 5/6, S, Inc | 2.2E-01 | 2.7E-01 | 0.78 |
|  |  | WeekTreatWeek 5/6, S, SA | -1.8E-01 | -2.1E-01 | 0.83 |
|  |  | WeekTreatWeek 8, M, Inc | -1.8E-01 | -1.8E-01 | 0.86 |
|  |  | WeekTreatWeek 8, M, SA | -1.7E+01 | -7.4E-03 | 0.99 |
|  |  | WeekTreatWeek 8, S, Inc | -1.8E-01 | -1.8E-01 | 0.86 |
| Phage capsid and scaffold protein CDS | AIG68656.1 | WeekTreatWeek 0, Control | -6.2E-18 | -1.4E-17 | 1.00 |
|  |  | WeekTreatWeek 3, M, Inc | -1.8E-01 | -2.9E-01 | 0.77 |
|  |  | WeekTreatWeek 3, M, SA | -4.1E-01 | -6.0E-01 | 0.55 |
|  |  | WeekTreatWeek 3, S, Inc | -1.8E-01 | -2.9E-01 | 0.77 |
|  |  | WeekTreatWeek 3, S, SA | -2.2E-01 | -3.3E-01 | 0.74 |
|  |  | WeekTreatWeek 5/6, M, Inc | -1.8E-01 | -3.3E-01 | 0.74 |
|  |  | WeekTreatWeek 5/6, M, SA | -1.8E-01 | -3.3E-01 | 0.74 |
|  |  | WeekTreatWeek 5/6, S, Inc | -8.7E-02 | -1.6E-01 | 0.87 |
|  |  | WeekTreatWeek 5/6, S, SA | -8.7E-02 | -1.6E-01 | 0.87 |
|  |  | WeekTreatWeek 8, M, Inc | -2.7E-17 | -4.4E-17 | 1.00 |
|  |  | WeekTreatWeek 8, M, SA | -1.8E-01 | -2.9E-01 | 0.77 |
|  |  | WeekTreatWeek 8, S, Inc | -1.8E-01 | -2.9E-01 | 0.77 |
| Hypothetical protein CDS | AIG68658.1 | WeekTreatWeek 0, Control | 1.4E-15 | 3.1E-15 | 1.00 |
|  |  | WeekTreatWeek 3, M, Inc | -1.5E-15 | -2.5E-15 | 1.00 |
|  |  | WeekTreatWeek 3, M, SA | -4.1E-01 | -6.0E-01 | 0.55 |
|  |  | WeekTreatWeek 3, S, Inc | -6.9E-01 | -9.5E-01 | 0.34 |
|  |  | WeekTreatWeek 3, S, SA | -9.2E-01 | -1.1E+00 | 0.27 |
|  |  | WeekTreatWeek 5/6, M, Inc | -6.9E-01 | -1.1E+00 | 0.25 |
|  |  | WeekTreatWeek 5/6, M, SA | -4.1E-01 | -7.1E-01 | 0.48 |
|  |  | WeekTreatWeek 5/6, S, Inc | -1.8E-01 | -3.3E-01 | 0.74 |
|  |  | WeekTreatWeek 5/6, S, SA | -4.1E-01 | -7.1E-01 | 0.48 |
|  |  | WeekTreatWeek 8, M, Inc | 2.9E-01 | 5.0E-01 | 0.61 |
|  |  | WeekTreatWeek 8, M, SA | -4.1E-01 | -6.0E-01 | 0.55 |
|  |  | WeekTreatWeek 8, S, Inc | -6.9E-01 | -9.5E-01 | 0.34 |
| Phage terminase small subunit CDS | AIG68659.1 | WeekTreatWeek 0, Control | 4.7E-01 | 1.3E+00 | 0.18 |
|  |  | WeekTreatWeek 3, M, Inc | -4.7E-01 | -8.7E-01 | 0.38 |
|  |  | WeekTreatWeek 3, M, SA | -1.8E-01 | -3.6E-01 | 0.72 |
|  |  | WeekTreatWeek 3, S, Inc | -1.8E-01 | -3.6E-01 | 0.72 |
|  |  | WeekTreatWeek 3, S, SA | -4.7E-01 | -8.2E-01 | 0.41 |
|  |  | WeekTreatWeek 5/6, M, Inc | -4.7E-01 | -1.0E+00 | 0.30 |
|  |  | WeekTreatWeek 5/6, M, SA | -3.9E-01 | -8.7E-01 | 0.39 |
|  |  | WeekTreatWeek 5/6, S, Inc | -3.2E-01 | -7.1E-01 | 0.48 |
|  |  | WeekTreatWeek 5/6, S, SA | -3.2E-01 | -7.1E-01 | 0.48 |
|  |  | WeekTreatWeek 8, M, Inc | -4.7E-01 | -8.7E-01 | 0.38 |
|  |  | WeekTreatWeek 8, M, SA | -3.2E-01 | -6.1E-01 | 0.54 |
|  |  | WeekTreatWeek 8, S, Inc | -1.8E-01 | -3.6E-01 | 0.72 |
| Putative Dnase CDS | AIG68660.1 | WeekTreatWeek 0, Control | -5.1E-01 | -8.8E-01 | 0.38 |
|  |  | WeekTreatWeek 3, M, Inc | 1.1E-01 | 1.4E-01 | 0.89 |
|  |  | WeekTreatWeek 3, M, SA | 5.1E-01 | 7.2E-01 | 0.47 |
|  |  | WeekTreatWeek 3, S, Inc | -1.8E-01 | -2.2E-01 | 0.82 |
|  |  | WeekTreatWeek 3, S, SA | -4.1E-01 | -4.4E-01 | 0.66 |
|  |  | WeekTreatWeek 5/6, M, Inc | 2.2E-01 | 3.3E-01 | 0.74 |
|  |  | WeekTreatWeek 5/6, M, SA | 2.2E-01 | 3.3E-01 | 0.74 |
|  |  | WeekTreatWeek 5/6, S, Inc | -2.8E-02 | -4.1E-02 | 0.97 |
|  |  | WeekTreatWeek 5/6, S, SA | -1.8E-01 | -2.6E-01 | 0.80 |
|  |  | WeekTreatWeek 8, M, Inc | 5.1E-01 | 7.2E-01 | 0.47 |
|  |  | WeekTreatWeek 8, M, SA | 5.1E-01 | 7.2E-01 | 0.47 |
|  |  | WeekTreatWeek 8, S, Inc | -1.8E-01 | -2.2E-01 | 0.82 |
| Phage tail assembly chaperone CDS | AIG68661.1 | WeekTreatWeek 0, Control | -2.2E+01 | -1.2E-03 | 1.00 |
|  |  | WeekTreatWeek 3, M, Inc | 3.2E-10 | 1.3E-14 | 1.00 |
|  |  | WeekTreatWeek 3, M, SA | 3.2E-10 | 1.3E-14 | 1.00 |
|  |  | WeekTreatWeek 3, S, Inc | 3.2E-10 | 1.3E-14 | 1.00 |
|  |  | WeekTreatWeek 3, S, SA | 3.2E-10 | 1.2E-14 | 1.00 |
|  |  | WeekTreatWeek 5/6, M, Inc | 2.0E+01 | 1.0E-03 | 1.00 |
|  |  | WeekTreatWeek 5/6, M, SA | 3.2E-10 | 1.4E-14 | 1.00 |
|  |  | WeekTreatWeek 5/6, S, Inc | 3.2E-10 | 1.4E-14 | 1.00 |
|  |  | WeekTreatWeek 5/6, S, SA | 3.2E-10 | 1.4E-14 | 1.00 |
|  |  | WeekTreatWeek 8, M, Inc | 3.2E-10 | 1.3E-14 | 1.00 |
|  |  | WeekTreatWeek 8, M, SA | 3.2E-10 | 1.3E-14 | 1.00 |
|  |  | WeekTreatWeek 8, S, Inc | 2.1E+01 | 1.1E-03 | 1.00 |
| Phage protein CDS | AIG68664.1 | WeekTreatWeek 0, Control | -1.2E-16 | -2.8E-16 | 1.00 |
|  |  | WeekTreatWeek 3, M, Inc | 3.5E-16 | 5.8E-16 | 1.00 |
|  |  | WeekTreatWeek 3, M, SA | 9.8E-17 | 1.6E-16 | 1.00 |
|  |  | WeekTreatWeek 3, S, Inc | 2.6E-16 | 4.3E-16 | 1.00 |
|  |  | WeekTreatWeek 3, S, SA | 1.4E-16 | 2.2E-16 | 1.00 |
|  |  | WeekTreatWeek 5/6, M, Inc | 2.7E-16 | 5.1E-16 | 1.00 |
|  |  | WeekTreatWeek 5/6, M, SA | 2.0E-16 | 3.7E-16 | 1.00 |
|  |  | WeekTreatWeek 5/6, S, Inc | -1.8E-01 | -3.3E-01 | 0.74 |
|  |  | WeekTreatWeek 5/6, S, SA | -8.7E-02 | -1.6E-01 | 0.87 |
|  |  | WeekTreatWeek 8, M, Inc | -6.6E-18 | -1.1E-17 | 1.00 |
|  |  | WeekTreatWeek 8, M, SA | -1.8E-01 | -2.9E-01 | 0.77 |
|  |  | WeekTreatWeek 8, S, Inc | 1.4E-17 | 2.3E-17 | 1.00 |
| Phage capsid and scaffold protein CDS | AIG68665.1 | WeekTreatWeek 0, Control | -2.2E-01 | -4.5E-01 | 0.66 |
|  |  | WeekTreatWeek 3, M, Inc | 4.1E-02 | 6.1E-02 | 0.95 |
|  |  | WeekTreatWeek 3, M, SA | 4.1E-02 | 6.1E-02 | 0.95 |
|  |  | WeekTreatWeek 3, S, Inc | 2.2E-01 | 3.5E-01 | 0.73 |
|  |  | WeekTreatWeek 3, S, SA | 5.2E-16 | 7.3E-16 | 1.00 |
|  |  | WeekTreatWeek 5/6, M, Inc | -6.5E-02 | -1.1E-01 | 0.91 |
|  |  | WeekTreatWeek 5/6, M, SA | 4.1E-02 | 6.9E-02 | 0.94 |
|  |  | WeekTreatWeek 5/6, S, Inc | 1.4E-01 | 2.3E-01 | 0.82 |
|  |  | WeekTreatWeek 5/6, S, SA | 4.1E-02 | 6.9E-02 | 0.94 |
|  |  | WeekTreatWeek 8, M, Inc | 4.1E-02 | 6.1E-02 | 0.95 |
|  |  | WeekTreatWeek 8, M, SA | 2.2E-01 | 3.5E-01 | 0.73 |
|  |  | WeekTreatWeek 8, S, Inc | 2.2E-01 | 3.5E-01 | 0.73 |
| Putative transport system permease protein CDS | AIG69104.1 | WeekTreatWeek 0, Control | 2.2E-17 | 4.8E-17 | 1.00 |
|  |  | WeekTreatWeek 3, M, Inc | -1.3E-17 | -2.2E-17 | 1.00 |
|  |  | WeekTreatWeek 3, M, SA | -1.3E-17 | -2.2E-17 | 1.00 |
|  |  | WeekTreatWeek 3, S, Inc | -1.3E-17 | -2.2E-17 | 1.00 |
|  |  | WeekTreatWeek 3, S, SA | -1.3E-17 | -2.1E-17 | 1.00 |
|  |  | WeekTreatWeek 5/6, M, Inc | -1.3E-17 | -2.5E-17 | 1.00 |
|  |  | WeekTreatWeek 5/6, M, SA | -1.3E-17 | -2.5E-17 | 1.00 |
|  |  | WeekTreatWeek 5/6, S, Inc | -1.3E-17 | -2.5E-17 | 1.00 |
|  |  | WeekTreatWeek 5/6, S, SA | -1.3E-17 | -2.5E-17 | 1.00 |
|  |  | WeekTreatWeek 8, M, Inc | -1.3E-17 | -2.2E-17 | 1.00 |
|  |  | WeekTreatWeek 8, M, SA | -1.3E-17 | -2.2E-17 | 1.00 |
|  |  | WeekTreatWeek 8, S, Inc | -1.3E-17 | -2.2E-17 | 1.00 |
| Hypothetical protein CDS | AIG69401.1 | WeekTreatWeek 0, Control | -1.6E+00 | -1.6E+00 | 0.11 |
|  |  | WeekTreatWeek 3, M, Inc | -1.8E+01 | -4.6E-03 | 1.00 |
|  |  | WeekTreatWeek 3, M, SA | -1.8E+01 | -4.6E-03 | 1.00 |
|  |  | WeekTreatWeek 3, S, Inc | 5.1E-01 | 4.2E-01 | 0.68 |
|  |  | WeekTreatWeek 3, S, SA | -1.8E+01 | -4.2E-03 | 1.00 |
|  |  | WeekTreatWeek 5/6, M, Inc | 5.1E-01 | 4.6E-01 | 0.65 |
|  |  | WeekTreatWeek 5/6, M, SA | -8.8E-01 | -6.2E-01 | 0.54 |
|  |  | WeekTreatWeek 5/6, S, Inc | -1.8E-01 | -1.5E-01 | 0.88 |
|  |  | WeekTreatWeek 5/6, S, SA | -1.8E-01 | -1.5E-01 | 0.88 |
|  |  | WeekTreatWeek 8, M, Inc | 5.1E-01 | 4.2E-01 | 0.68 |
|  |  | WeekTreatWeek 8, M, SA | -1.8E-01 | -1.3E-01 | 0.90 |
|  |  | WeekTreatWeek 8, S, Inc | -1.8E-01 | -1.3E-01 | 0.90 |
| Hypothetical protein CDS | AIG69967.1 | WeekTreatWeek 0, Control | -2.0E+01 | -2.9E-03 | 1.00 |
|  |  | WeekTreatWeek 3, M, Inc | -1.0E-10 | -1.1E-14 | 1.00 |
|  |  | WeekTreatWeek 3, M, SA | 1.9E+01 | 2.7E-03 | 1.00 |
|  |  | WeekTreatWeek 3, S, Inc | -1.0E-10 | -1.1E-14 | 1.00 |
|  |  | WeekTreatWeek 3, S, SA | -1.0E-10 | -1.1E-14 | 1.00 |
|  |  | WeekTreatWeek 5/6, M, Inc | -1.0E-10 | -1.3E-14 | 1.00 |
|  |  | WeekTreatWeek 5/6, M, SA | 1.9E+01 | 2.7E-03 | 1.00 |
|  |  | WeekTreatWeek 5/6, S, Inc | 1.9E+01 | 2.7E-03 | 1.00 |
|  |  | WeekTreatWeek 5/6, S, SA | 1.9E+01 | 2.7E-03 | 1.00 |
|  |  | WeekTreatWeek 8, M, Inc | 1.9E+01 | 2.7E-03 | 1.00 |
|  |  | WeekTreatWeek 8, M, SA | -1.0E-10 | -1.1E-14 | 1.00 |
|  |  | WeekTreatWeek 8, S, Inc | 1.9E+01 | 2.7E-03 | 1.00 |
| GTP-binding protein TypA/BipA CDS | AIG71329.1 | WeekTreatWeek 0, Control | -2.1E+01 | -1.9E-03 | 1.00 |
|  |  | WeekTreatWeek 3, M, Inc | 2.0E+01 | 1.7E-03 | 1.00 |
|  |  | WeekTreatWeek 3, M, SA | 2.0E+01 | 1.8E-03 | 1.00 |
|  |  | WeekTreatWeek 3, S, Inc | 5.1E-10 | 3.3E-14 | 1.00 |
|  |  | WeekTreatWeek 3, S, SA | 5.1E-10 | 3.1E-14 | 1.00 |
|  |  | WeekTreatWeek 5/6, M, Inc | 1.9E+01 | 1.6E-03 | 1.00 |
|  |  | WeekTreatWeek 5/6, M, SA | 5.1E-10 | 3.7E-14 | 1.00 |
|  |  | WeekTreatWeek 5/6, S, Inc | 5.1E-10 | 3.7E-14 | 1.00 |
|  |  | WeekTreatWeek 5/6, S, SA | 1.9E+01 | 1.6E-03 | 1.00 |
|  |  | WeekTreatWeek 8, M, Inc | 5.1E-10 | 3.3E-14 | 1.00 |
|  |  | WeekTreatWeek 8, M, SA | 5.1E-10 | 3.3E-14 | 1.00 |
|  |  | WeekTreatWeek 8, S, Inc | 5.1E-10 | 3.3E-14 | 1.00 |
