# Supplementary material for: Absence of genetic selection in a pathogenic Escherichia coli strain exposed to the manure-amended soil environment
Source: PLoS One. 2018 Dec 7;13(12):e0208346. doi: 10.1371/journal.pone.0208346 (PMC6286177; doi:10.1371/journal.pone.0208346)
Supplement: S4 Table — The MG-RAST predicted features used to annotate reads were: the top 25 genuses (by frequency) and functional subsystems, except for those that were correlated with all other functional subsystems. Subsequently, all p values were then ordered and compared to threshold values calculated using the Benjamini-Hochberg (BH) procedure, to account for multiple comparisons. The false discovery rate (α) was set at 0.25 to ensure no possible significant factors were overlooked. A p value less than the corresponding threshold is considered significant. (DOCX) [file pone.0208346.s004.docx]

| **Response variable** | ***P* value** | **BH Threshold** |
| --- | --- | --- |
| Yersinia | 0.11 | 0.01 |
| Phages, Prophages, Transposable elements, Plasmids | 0.17 | 0.01 |
| Salmonella | 0.25 | 0.02 |
| Nitrogen Metabolism | 0.31 | 0.03 |
| Shigella | 0.31 | 0.03 |
| Enterobacter | 0.33 | 0.04 |
| Escherichia | 0.38 | 0.05 |
| Respiration | 0.40 | 0.05 |
| Phosphorus Metabolism | 0.41 | 0.06 |
| Bacteria | 0.41 | 0.07 |
| Iron acquisition and metabolism^1^ | 0.31 | 0.07 |
| Stress Response | 0.48 | 0.08 |
| Pectobacterium | 0.51 | 0.09 |
| Dormancy and Sporulation^2^ | 0.54 | 0.09 |
| Serratia | 0.52 | 0.10 |
| Lambda like viruses | 0.55 | 0.11 |
| Viruses | 0.56 | 0.11 |
| Ruminococcus | 0.59 | 0.12 |
| Cronobacter | 0.60 | 0.13 |
| Bacteroides | 0.61 | 0.14 |
| Populus | 0.61 | 0.14 |
| Erwinia | 0.62 | 0.15 |
| Citrobacter | 0.66 | 0.16 |
| Klebsiella | 0.68 | 0.16 |
| Gallus | 0.72 | 0.17 |
| Pseudomonas | 0.73 | 0.18 |
| Streptococcus | 0.74 | 0.18 |
| Metabolism of Aromatic Compounds^1^ | 0.84 | 0.19 |
| Potassium metabolism | 0.79 | 0.20 |
| Motility and Chemotaxis^1^ | 0.89 | 0.20 |
| Vibrio^2^ | 0.76 | 0.21 |
| Haemophilus | 0.83 | 0.22 |
| Acinetobacter | 0.84 | 0.22 |
| Neisseria | 0.85 | 0.23 |
| Drosophila | 0.87 | 0.24 |
| Propionibacterium | 0.90 | 0.24 |
| Mycobacterium^2^ | 0.90 | 0.25 |

^1^Data were log transformed

^2^Data were square-root transformed
