# Supplementary material for: Absence of genetic selection in a pathogenic Escherichia coli strain exposed to the manure-amended soil environment
Source: PLoS One. 2018 Dec 7;13(12):e0208346. doi: 10.1371/journal.pone.0208346 (PMC6286177; doi:10.1371/journal.pone.0208346)
Supplement: S5 Table — The location of each regulator in the E. coli O157:H7 EDL933 reference genome used in this study is provided (GenBank accession no. CP008957.1) [24], along with an indication of whether a gap or SNP was found in the regulator through mapping to the reference genome on CLC Workbench. (DOCX) [file pone.0208346.s005.docx]

| **Regulator** | **Genbank accession number** | **Location in reference genome** | **CLC gap?** | **CLC SNP?** |
| --- | --- | --- | --- | --- |
| RpoS | AIG70067.1 and AIG70068.1 | 3674708 – 3674938 and 3674978 – 3675700 | No | No |
| Curlin genes transcriptional activator (Crl) | AIG66479.1 | 295717 – 296118 | No | No |
| CsgD | AIG67802.1 | 1549421 – 1550071 | No | No |
| MlrA | AIG69446.1 | 3032141 – 3032788 | No | No |
| Cra (FruR) | AIG66291.1 | 92634 – 93638 | No | No |
| Cyclic AMP receptor protein (Crp) | AIG70709.1 | 4293653 – 4294285 | No | No |
| Transcriptional regulator TetR family (RcdA) | AIG67174.1 | 1012481 – 1013017 | No | No |
| Integration host factor (IHF) | AIG67370.1 and AIG68843.1 | 1180803 – 1181087 and 2489428 – 2489727 | No | No |
| DNA binding protein H-NS | AIG68128.1 | 1822740 – 1823153 | No | No |
| CpxA/R | AIG71379.1 and AIG71380.1 | 4991188 – 4992561; 4992558 – 4993190 | No | No |
| EnvZ/OmpR | AIG70752.1 and AIG70753.1 | 4334337 – 4335689; 4335686 – 4336405 | No | No |
| Sensory histidine kinase in two-component regulatory system (RstB)/RstA | AIG68735.1 and AIG68734.1 | 2377144 – 2378445; 2376421 – 2377140 | No | No |
| ArcA/B | AIG71882.1 and AIG70586.1 | 545261 – 5545977; 4180669 – 4183005 | No | No |
| BasS/R | AIG71596.1 and AIG71597.1 | 5235496 – 5236596; 5236597 – 5237265 | No | No |
| OmrA/B | NA | *3786128-3786215; *3786331 - 3786412 | No | No |
| McaS | NA | *2181172 – 2181337 | No | No |
| GcvB | NA | *3752722 – 3752927 | No | No |
| RprA | NA | *2464545 – 2464650 | No | No |
| ArcZ | NA | *4180557 – 4180677 | No | No |
| SdsR | NA | *2619457 – 2619577 | No | No |

*Indicates that the gene annotation was not annotated in EDL933, but a match was found after BLASTing the *E. coli* K12 segment against EDL933.
