# Supplementary figures and images for: Absence of genetic selection in a pathogenic Escherichia coli strain exposed to the manure-amended soil environment
Source: PLoS One. 2018 Dec 7;13(12):e0208346. doi: 10.1371/journal.pone.0208346 (PMC6286177; doi:10.1371/journal.pone.0208346)

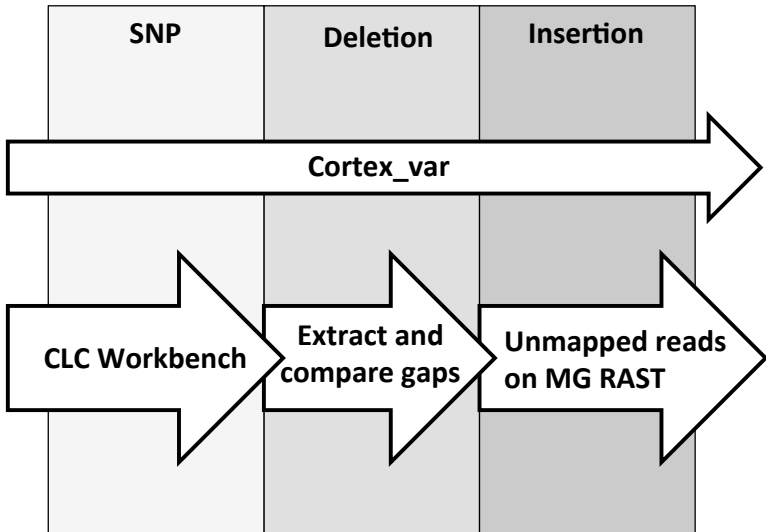

Supplement: S1 Fig — (PDF) [file pone.0208346.s006.pdf]

**Curli.phenotype**

Red  
Mixed

White

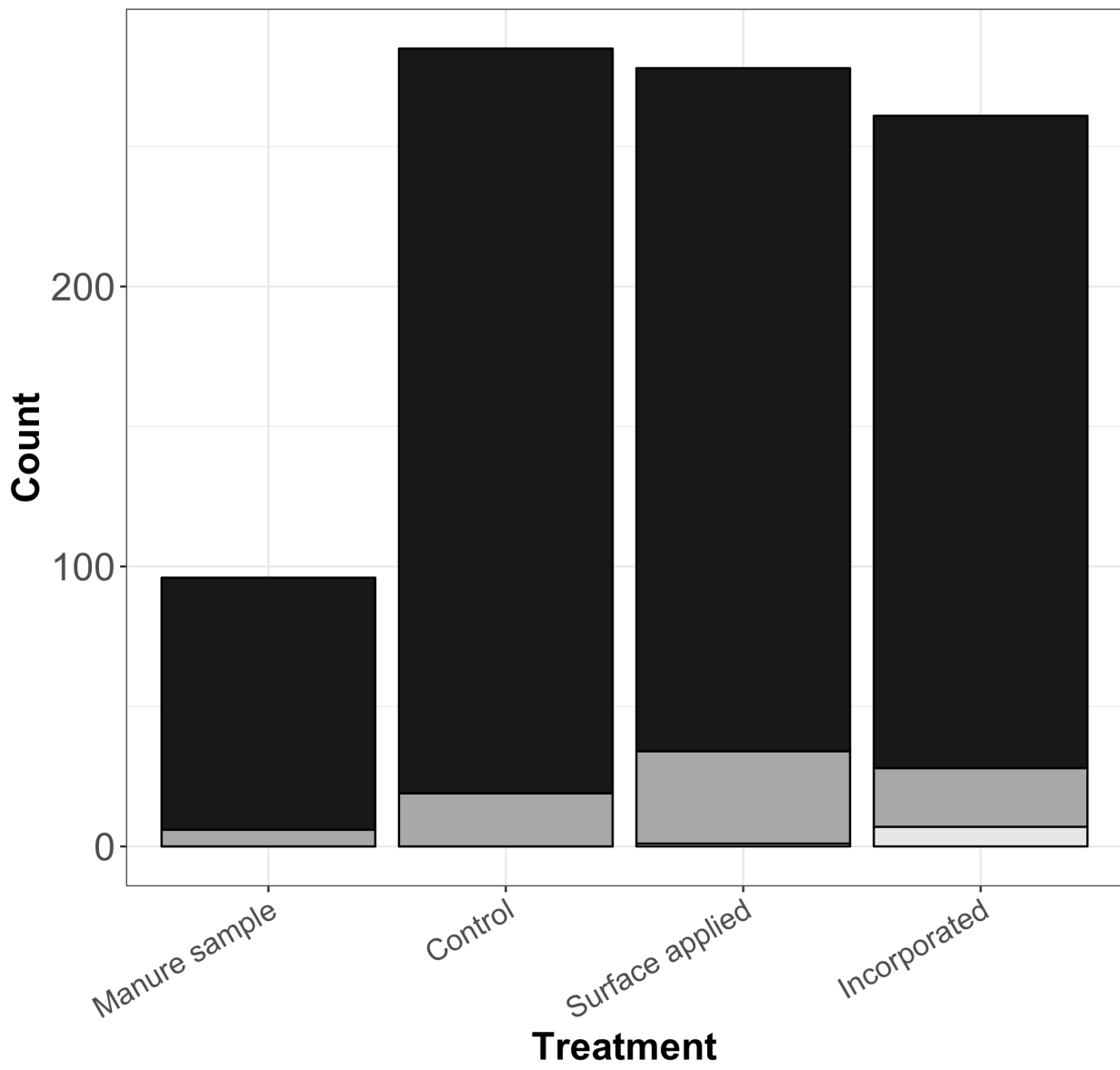

Supplement: S2 Fig — The ratio of isolates with a mixed red and white morphotype to isolates with a red morphotype was highest in the population sampled from the top 2 cm of soil in the surface applied treatment column. A red colony morphotype on CR agar indicates curli production, while a white morphotype indicates no curli production. For all treatments, the red morphotype was the predominant morphotype in the population. Control treatments had no added manure, incorporated treatments mixed the manure into the top 5 cm of soil, and surface-applied treatments left the manure on the soil surface. The manure sample was taken from the manure used in all treatments. (PDF) [file pone.0208346.s007.pdf]

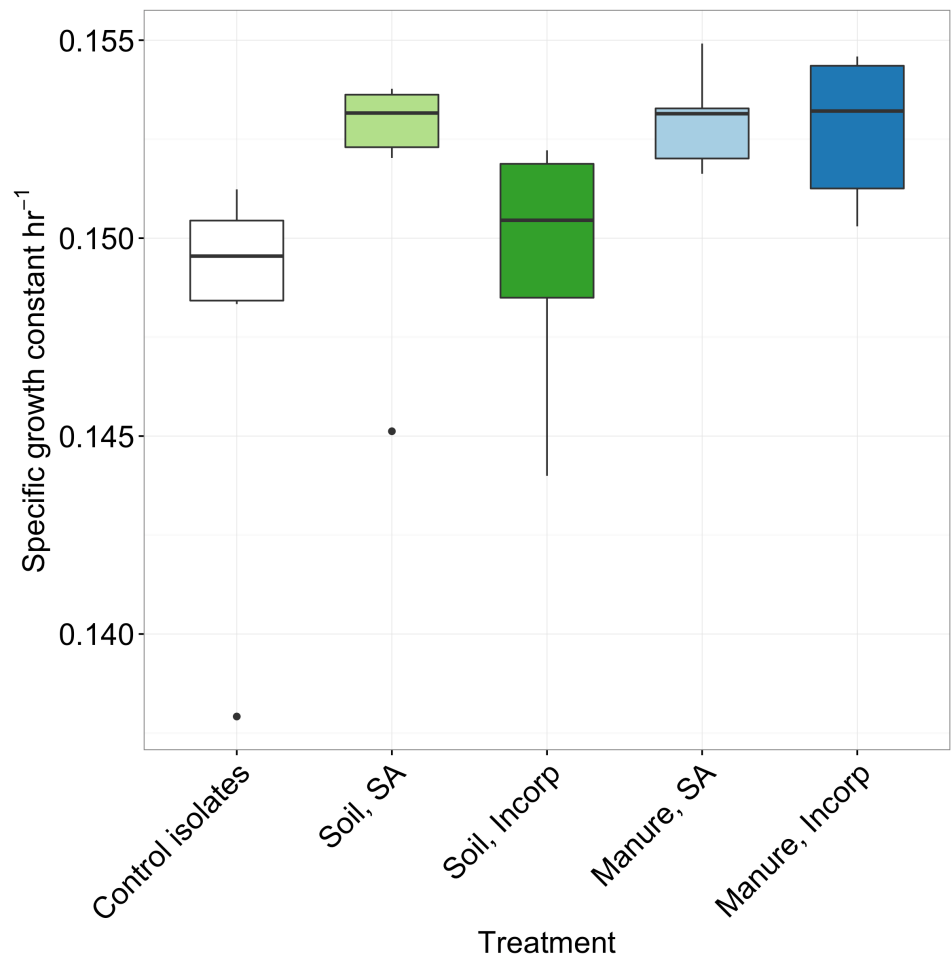

Supplement: S3 Fig — Collected from each treatment type at week 6, and the control (week 0). Grown over a 14-hr period in LB broth at 37°C. Data represents six replicates for each treatment. (PDF) [file pone.0208346.s008.pdf]

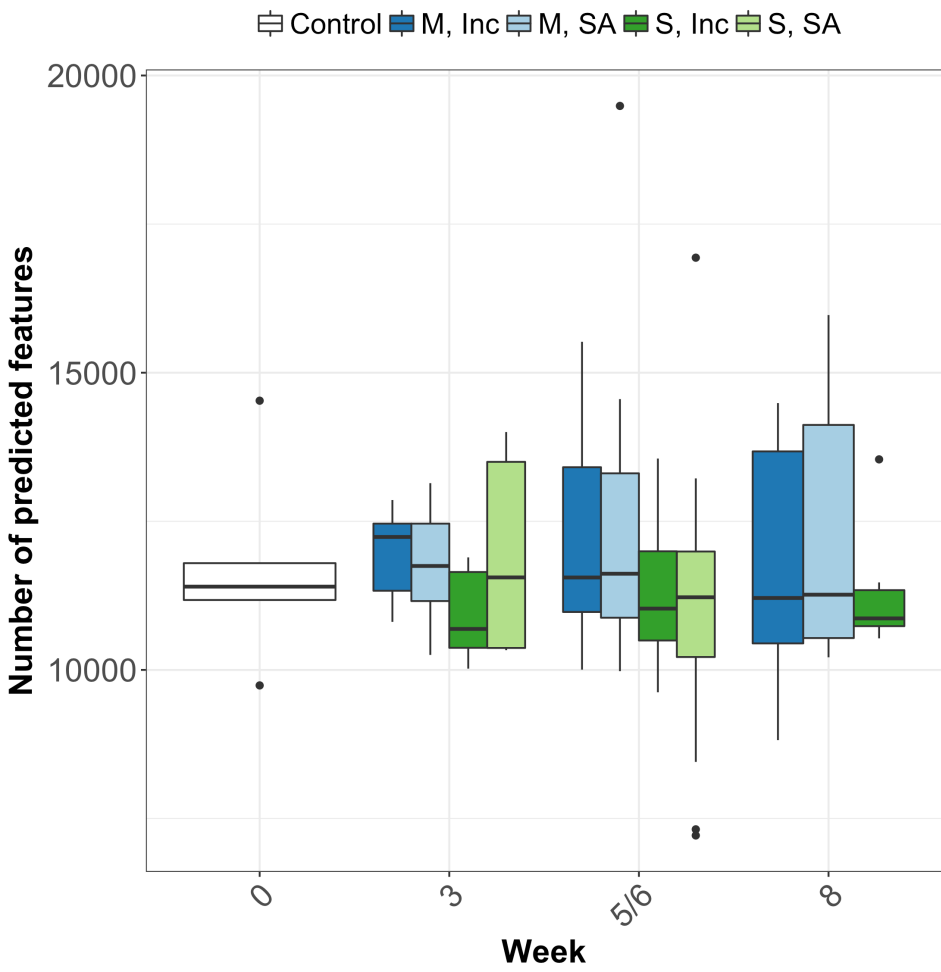

Supplement: S4 Fig — The reads are plotted by both the week the isolate was collected and the soil-manure treatment to which the isolate was exposed. Four points with values greater than 25,000 were excluded to make the plot easier to read. These points came from week 3 soil-incorporated, week 5/6 manure-incorporated, week 5/6 manure surface-applied, and week 8 manure-incorporated strains. (PDF) [file pone.0208346.s009.pdf]
